# Antineoplastic potential of *Lepisanthes rubiginosa* (Roxb.) Leenh. leaves extract against ehrlich ascites carcinoma: A multi‒approach study

**DOI:** 10.1016/j.bbrep.2026.102835

**Published:** 2026-10-20

**Authors:** Md. Taohid Hasan, Md. Shakil Ahmed, Niloy Saha, Reaz Ahmmed, Md. Enamul Kabir Talukder, Nafis Fuad Shahir, Nazia Islam Rafi, Farzana Pervin, Masudul Hasan Khan, Md. Rezaul Karim, Mohammad Amirul Islam

**Affiliations:** a Department of Biochemistry and Molecular Biology, Faculty of Science, University of Rajshahi, Rajshahi, 6205, Bangladesh; b Institute of Biological Sciences (IBSc), University of Rajshahi, Rajshahi, 6205, Bangladesh; c Department of Genetic Engineering and Biotechnology, Faculty of Biological Science and Technology, Jashore University of Science and Technology, Jashore, 7408, Bangladesh

**Keywords:** *Lepisanthes rubiginosa*, Phytopharmaceutical potential, Antioxidant activity, Antineoplastic activity, Molecular docking, MD simulations

## Abstract

This study evaluated the antineoplastic potential of *L. rubiginosa* leaves by integrating phytochemical, antioxidant, *in vivo*, and *in silico* approaches. The methanolic extract of *L. rubiginosa* leaves (LRLME) contained high levels of phenolics (61.292 ± 0.902 mg GAE/g) and flavonoids (130.718 1.862 mg CAE/g) and exhibited strong antioxidant activity in the total antioxidant capacity, DPPH, and ABTS assays. Cytotoxicity assessment using the brine shrimp lethality assay indicated low toxicity, with an LC_50_ of 661.278 μg/mL. *In vivo* antitumor studies revealed that intraperitoneal administration of LRLME (40 mg/kg) significantly inhibited Ehrlich Ascites Carcinoma (EAC) cell proliferation by 54.31% (p < 0.001) and extended the lifespan of mice implanted with tumors by 97.30% (p < 0.001). Treatment with LRLME also significantly restored hematological parameters, including red and white blood cell counts and hemoglobin levels, toward normal values compared with untreated controls. Gas chromatography–mass spectrometry (GC–MS) profiling revealed 54 phytochemical constituents, which were thereafter screened through molecular docking, ADMET profiling, and molecular dynamics simulations targeting the epidermal growth factor receptor (EGFR). Three compounds, namely (−)-norephedrine, cathine, and phenylephrine, demonstrated the highest binding affinities (−6.644, −6.474, and −6.000 kcal/mol, respectively) and favorable pharmacokinetic and toxicity profiles. Molecular dynamics studies further affirmed the sustained stable interactions and conformational stability of these compounds within the EGFR active site. Collectively, these findings suggest that *L. rubiginosa* exhibits significant anticancer activity against EAC cells and may serve as a potential natural reservoir of EGFR inhibitors for future therapeutic development.

## Highlights

• *L. rubiginosa* leaf extract showed strong antioxidant activity and high polyphenols.
• The extract inhibited EAC growth and improved survival in tumor-bearing mice.
• The extract restored altered hematological parameters toward normal.
• GC–MS identified 54 phytocompounds; three lead showed strong EGFR protein binding.
• MD simulation confirmed CID 441457 (cathine) as the most stable EGFR binder.

## Graphical abstract

**Figure:**
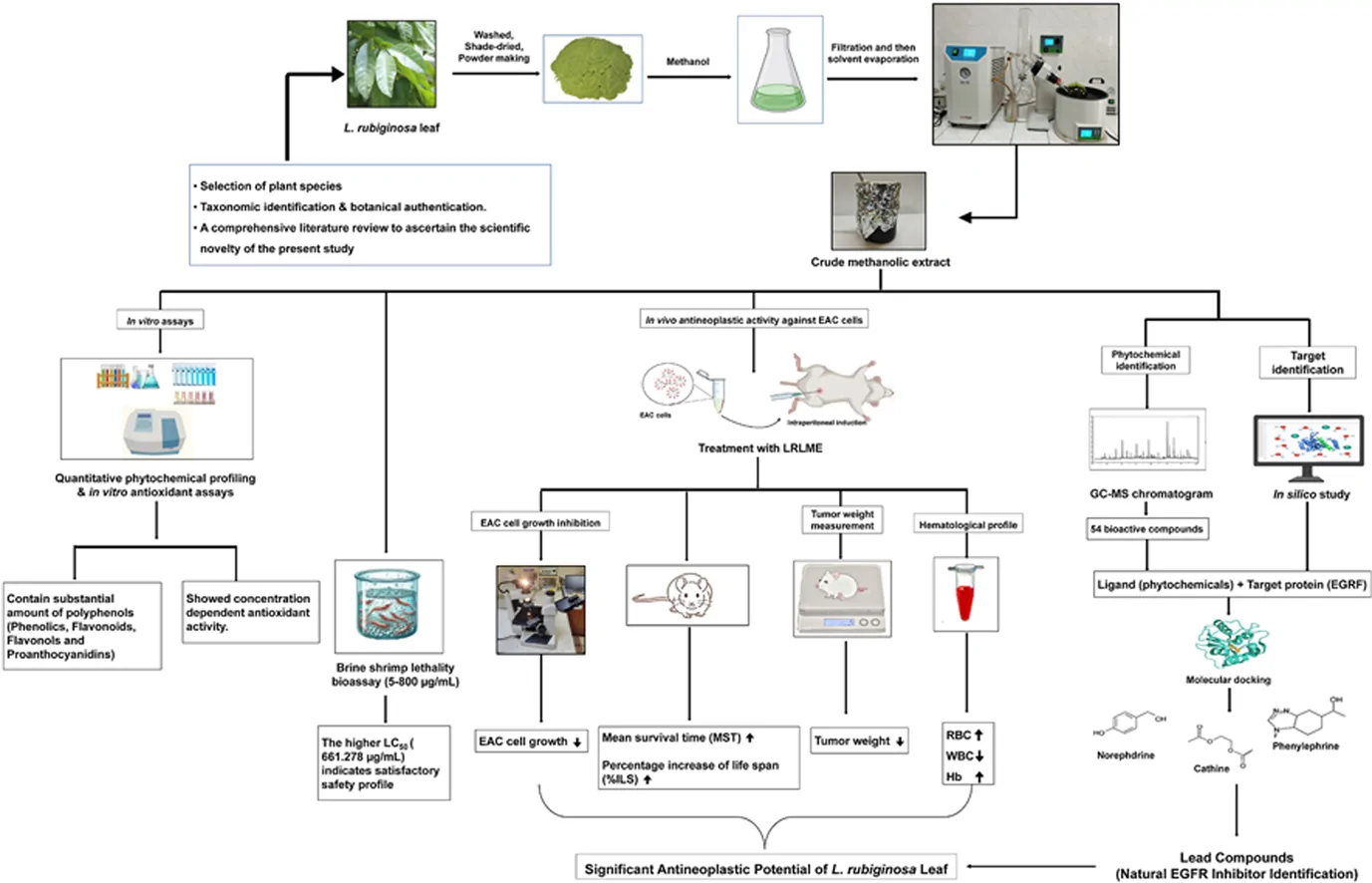

## 1 Introduction

Cancer has established itself as one of the foremost health threats globally, exhibiting a marked increase in occurrence across human populations [1]. Signal transduction pathways are pivotal in maintaining cellular homeostasis. However, during cancer development, successive genomic and epigenetic modifications facilitate the evasion of these homeostatic controls, resulting in dysregulated and sustained cellular proliferation [2]. As per the Global Cancer Statistics 2024 report, an estimated 20 million individuals were newly confirmed with cancer in 2022, as well as roughly 9.7 million lives were lost to the disease. If current trends persist, the global cancer incidence is expected to escalate to 35 million new cases per year by 2050 [3].

We are constantly exposed to a variety of physical and chemical dangers in our daily lives, such as food additives, ionizing and ultraviolet (UV) radiation, and environmental pollution. Apart from these external stressors, free radical formation and reactive oxygen species (ROS) generation originate from intrinsic cellular activities and pathological stimuli, largely centered in endoplasmic reticulum (ER) compartments, peroxisomes, in conjunction with mitochondria [4]. ROS generation is considered a critical contributor in oncogenic processes, with numerous studies highlighting its roles in cellular transformation, genomic instability, and tumor progression [5]. Damage to DNA bases and the deoxyribose backbone by ROS/RNS (reactive nitrogen species) results in severe alterations in fundamental genes that trigger the initiation, promotion, and progression phases of carcinogenesis [5]. Furthermore, free radicals have been shown to promote tumorigenesis by activating intracellular signaling pathways that trigger the activation of proto-oncogenic transcription factors, namely c-MYC, c-FOS, and c-JUN, thereby facilitating the uncontrolled proliferation of cancer cells [6].

Ehrlich Ascites Carcinoma (EAC) cells are undifferentiated, transplantable murine tumor cells derived from a spontaneous mammary adenocarcinoma in mice. When inoculated intraperitoneally, EAC cells proliferate rapidly within the peritoneal cavity, leading to ascites formation and systemic malignancy. EAC cells possess some characteristics, like a high proliferation rate, abnormal upregulation of BCL-2 family anti-apoptotic proteins, secretion of angiogenic factors such as VEGF supporting neovascularization, redox imbalance, and suppression of host immune responses, so that they can be suitably used in cancer models [7]. EGFR functions as a cell-surface glycoprotein receptor embedded in the membrane and is part of the ErbB tyrosine kinase family, mediating key signaling pathways involved in cellular growth and differentiation. The binding of epidermal growth factor (EGF) to EGFR facilitates homo- or heterodimerization of the receptor, activating its intrinsic catalytic kinase domain, promoting self-catalyzed phosphorylation at crucial tyrosine residues, and facilitating signal transmission through downstream cascades, notably the STAT pathways, PI3K/Akt, and MAPK. These recruited signaling complexes include the RAS-RAF-MEK-ERK cascade, which promotes proliferation, the PI3K/Akt pathway, which regulates survival alongside anti-apoptotic responses, and the STAT pathway, which controls gene expression and cell growth. Aberrant EGFR signaling due to overexpression and mutations (e.g., exon 19 and 21 L858R) is linked to several cancer progression processes. Furthermore, EGFR activation contributes to tumor progression by inducing VEGF-mediated angiogenesis and promoting epithelial–mesenchymal transition (EMT), which collectively enhance metastatic dissemination and tissue invasion [8]. EGFR lies at the frontline of these cellular signaling networks, making it a more favorable therapeutic target compared to downstream signaling components. Its location on the cell surface, combined with its status as a druggable kinase that is frequently overexpressed and genetically altered in various cancers, further underscores its attractiveness as a target for cancer therapy [9].

Current methods of cancer treatment include chemotherapy, radiotherapy, immunotherapy, surgery, molecular targeted therapy, hormone replacement therapy, etc. Chemotherapy is the most commonly used cancer treatment method; however, its effectiveness is decreased by its side effects, including significant toxicity, inactivity, multidrug resistance, and high cost [10], [11]. Consequently, new approaches for preventing anticancer drug resistance are required. In the context of modern research efforts, considerable attention has centered on discovering natural agents that are able to neutralize free radicals/ROS [12]. Many antioxidants exhibit cancer-preventing or anticarcinogenic effects [12], [13]. Recent research has indicated that antioxidant molecules, which are found in most plants, may have numerous positive effects on human health. Plant-derived antioxidants attenuate oxidative damage through free radical quenching, suppressing oxidative processes, concurrently enhancing the activity of endogenous antioxidant systems. Consequently, it has been suggested to eat more herbal plants, which may be crucial in preventing illnesses linked to oxidative stress and maintaining a healthy status [14]. The therapeutic superiority of natural compounds compared to synthetic drugs is particularly due to their inherent aptitude to bind to the multi-drug targets like cellular receptors, signaling molecules, proteins, and nucleic acids. Interestingly, phytocompounds are likely to bind with multi-target sites through a wide range of chemical spaces but without off-target cytotoxic effects [15]. From 1981 to 2019, approximately 50% of FDA-approved drugs were of natural origin or mimetics of natural substances [16]. Phytocompounds have been extensively investigated as potential candidates in cancer treatment, aiding in the exploration of innovative pharmacologically active candidates. The exploration of novel anticancer leads, for which phytocompounds might represent an appropriate source, is a core focus of scientists and pharmaceutical developers throughout the world [17]. Traditional plant-based therapies continue to serve as the primary healthcare resource for over 80% of the global population (WHO) [18]. Finding plant species with potential pharmaceutical characteristics is therefore a crucial research topic, facilitating the discovery of new drug candidates. Plant-based compounds like polyphenols, terpenoids, and alkaloids have the potential to revolutionize cancer treatment and can influence multiple cellular processes, making them promising candidates for developing targeted therapies with broad-spectrum effects [19]. It has been demonstrated that these phytobioactive substances concurrently alter enzyme-catalyzed processes, transduction mediators, integrated with receptor-mediated cellular responses, suggesting that they could have possess a variety of biological targets, making them potentially therapeutic with several targets and components involved [17], [20].

*L. rubiginosa*, locally known as “Chagalnadi,” is a small evergreen tree or shrub from the Sapindaceae family, commonly growing 2–3 m, with potential to reach 7 m. It is detected in village thickets and almost all forest areas of Bangladesh, as well as in South and Southeast Asia and Australia [21], [22], [23]. This plant's various parts have a long-standing record of use in classical, Ayurvedic, and Unani healthcare formulation owing to their medicinal potential in alleviating against diverse disease manifestations such as pruritus, headache, fever, muscle soreness, flatulence, postpartum blues, as a decoction, facial skin problems, diarrhea, dysentery, jaundice, insomnia, cough, and as a tonic and food preservative [23], [24], [25]. A comprehensive literature review on *L. rubiginosa* revealed that different types of extracts of *L. rubiginosa*'s different parts (leaves, fruits, bark, seeds, roots) possess bioactive alkaloids, polyphenolic constituents, saponins, glycosides, tannins, and sterol derivatives [26]. It is found that different parts of *L. rubiginosa* exhibited antimicrobial, antioxidant, antimalarial, analgesic, antidiarrheal, antihyperglycemic, antipyretic, and neuropharmacological activities [27], [28]. The butanol extract of this plant isolated 3-O-[β-d-xylopyranosyl-(1 → 3)-β-d-glucopyranosyl]-oleanolic acid that exhibited appreciable anticancer activity across four malignant cell lines [29]. Research on Vietnamese plant extracts revealed that the alcoholic extract of *L. rubiginosa* bark exhibited nonspecific toxicity in a murine leukemia model (P388) that was given *in vivo* at 40 mg/kg, coupled with *in vitro* antiproliferative effects on A549 lung carcinoma cells at concentrations between 10 and 30 μg/mL [30].

The drug design and development methodologies are remarkably transformed from traditional techniques to the integration of computational techniques, notably computer-aided drug design (CADD) [17], [31]. Traditional drug design and lead optimization require long‒term assessment with lower precision. In contrast, CADD has been a well-recognized and reliable strategy for more than three decades, extensively applied in the identification, optimization, and development of potential pharmacologically active molecular lead compounds. *In silico* docking, ADME studies, toxicity screening, and molecular dynamics-based computational analysis are the key approaches used to explore lead candidates from vast libraries promptly [32]. The affinity score of ligands toward target proteins can be effectively assessed using molecular docking techniques. Additionally, molecular dynamics (MD) simulations create an artificial environment that simplifies the screening process by evaluating the stable association of ligand and protein throughout the simulation timeframe. The *in silico* prediction of pharmacokinetic properties and toxicity profiles demonstrates whether candidate drugs can effectively reach the target sites and helps identify potential adverse health effects [33], [34]. Therefore, a comprehensive literature review revealed that the *L. rubiginosa* plant possesses diverse pharmacologically active compounds and has remarkable biological potential. Although a preliminary phytochemical characterization of *L. rubiginosa* leaf has recently been reported [35], no previous reports have detailed a study on antioxidant properties and antineoplastic activity, along with *in silico* molecular lead identification from GC-MS results. Moreover, no prior *in vivo* studies have evaluated the biological activities of the plant's leaf sample using a suitable animal model. Therefore, the present investigation was designed with three main objectives. First, to characterize the phytochemical composition of the methanolic extract of *L. rubiginosa* leaf through spectrophotometric and gas chromatography-mass spectrometry assays as a basis for subsequent biological and computational investigations. Second, to explore the *in vitro* antioxidant capacity of *L. rubiginosa* extract and to assess its *in vivo* antineoplastic effects, aiming to elucidate its therapeutic potential in cancer management. Third, to identify drug-like substances from GC-MS data by using computational studies, including molecular docking and MD simulations with the EGFR target protein. By scientifically validating the biological activities of this traditionally used medicinal plant and identifying promising bioactive constituents, this work may facilitate future natural product-based anticancer drug discovery and support the development of safer and more effective therapeutic strategies against cancer.

## 2 Study design and analytical methods

### 2.1 Reagents and materials employed

Reagents such as DMSO (dimethyl sulfoxide), gallic acid, Folin–Ciocalteu's phenol reagent, sodium hydroxide, catechin, AlCl_3_, Na_2_CO_3_, ABTS•+, and DPPH• utilized in the current investigation were sourced from Sigma-Aldrich (St. Louis, MO, USA). Scharlau, Spain, was the provider of ascorbic acid (AA). The supplier of potassium persulfate (K_2_S_2_O_8_) was Wako Pure Chemical Industries Ltd., Japan. E-Mark (Germany) delivered the methanol and chloroform. Analytical-grade chemicals were employed in this research. Whatman No. 1 filter paper (Grade-1, England) was employed to make the crude extract of the chosen sample. Distilled water was utilized for preparing standards.

### 2.2 Plant sampling and extraction procedures

During the late winter season of February 2024, leaves of *L. rubiginosa* were gathered from the Sirajganj region (Ghurka, Raiganj, Salanga, Sirajganj; GPS coordinates: 24° 24′ 57″ N Latitude, 89° 30′ 27″ E Longitude) of the Rajshahi Division, Bangladesh. Plant identification and authentication were carried out by the eminent taxonomist Dr. A.H.M. Mahbubur Rahman, a Professor of the Department of Botany at the University of Rajshahi. A voucher specimen with an Accession Number 389 has been deposited in the herbarium under the Department of Botany of the university for future reference and taxonomic validation. After collection, *L. rubiginosa* leaves were cleaned thoroughly under flowing water to eliminate soil and surface contaminants, then air-dried in the shade at room temperature (RT) for 12 days. The dried plant material was coarsely powdered by using a grinder (FFC-15, China) and preserved in vacuum-sealed containers at RT. For extraction, 150 g of the dried powdered sample was macerated in 600 mL of methanol (1:4 w/v) in a round-bottom bottle at RT for 15 days with intermittent shaking. The extract was filtered via Whatman No. 1 filter paper, and the filtrate was concentrated at 39 °C and 261 mbar vacuum pressure employing a rotary evaporator (LabTech EV400 VAC, California, USA) to obtain the crude methanolic extract, designated as LRLME. The crude extract was maintained in sealed containers (4 °C) for subsequent analyses.

### 2.3 Polyphenol quantification of LRLME

#### 2.3.1 Total phenolics estimation in LRLME

A modified Folin–Ciocalteu method, based on Hossain and Rahman [36], was conducted to assess the phenolic content in LRLME. The procedure involved mixing 0.3 mL of the extract (1 mg/mL) with 2.5 mL of the Folin–Ciocalteu reagent (diluted 1:10), then 2.25 mL of 6% Na_2_CO_3_ solution was added after incubating for 5 min at RT. After mixing thoroughly, the samples were incubated for 90 min at RT to ensure proper color formation. Absorbance was recorded at 650 nm by using a UV-VIS spectrophotometer (DRAWELL DU8200-202503DR005, Shanghai, China). The total phenolic content was derived from a gallic acid standard and expressed as mg GAE/g of dry extract.

#### 2.3.2 Estimation of flavonoids in LRLME

With minor adjustments, the AlCl_3_‒colorimetric workflow was operated to quantify the total flavonoid level of the crude extract [37]. Briefly, A 0.5 mL portion of the extract solution (1 mg/mL) was introduced into 0.15 mL of a sodium nitrite solution (5%), and the final volume was adjusted to 1 mL by utilizing methanol, followed by dilution with 2.25 mL of distilled water. After standing for 6 min at RT, 0.3 mL of an aluminum chloride hexahydrate solution (10%) was poured and left to react for 5 min. Subsequently, 1 mL of sodium hydroxide (1 M) was introduced, and the reaction mixture was incubated for 15 min before recording absorbance at 430 nm on a UV–VIS spectrophotometer. Precise determination was achieved by applying a catechin calibration curve, and the outcomes were presented as milligrams of catechin equivalents (CAE) per gram of extract on a dry weight basis.

#### 2.3.3 Estimation of flavonols in LRLME

Flavonol content determination in the extract was accomplished following the colorimetric practice of Kumaran and Karunakaran (2007) [38]. For each assay, 2.0 mL of the extract or standard solution (1 mg/mL) was assembled with 3.0 mL of sodium acetate (50 g/L) and 2.0 mL of 2% aluminum chloride solution dissolved in ethanol. After an incubation period of 2.5 h at 20 °C, the optical density at 440 nm was determined via a UV–VIS spectrophotometer. The total flavonol content was derived from a quercetin standard and expressed as mg QUE/g of dry extract.

#### 2.3.4 Estimation of proanthocyanidins in LRLME

Proanthocyanidin content was determined as per the workflow of Ahmed et al. (2025) [39]. In this assay, 0.5 mL extract/standard (1 mg/mL) was admixed with 3.0 mL 4% vanillin in methanol and 1.5 mL HCl (concentrated). The reaction mixture was incubated for 15 min, after which the optical density at 500 nm was measured. Precise determination was achieved by applying a catechin calibration curve, and the outcomes were presented as mg CAE/g of LRLME on a dry weight basis.

### 2.4 *In vitro* screening of antioxidant potency

#### 2.4.1 Assessment of antioxidant capacity (TAC assay)

With slight adjustments, the phosphomolybdenum technique [36] was used for determining the total antioxidant capacity of LRLME. To put it briefly, 0.3 mL of the leaf extract, prepared at varying concentrations, was combined with 3 mL of a mixture composed of 0.6 M H_2_SO_4_, 28 mM sodium phosphate, and 4 mM ammonium heptamolybdate ((NH_4_)_6_Mo_7_O_24_). The reaction was carried out at 95 °C for 90 min. Following permitted to reach typical laboratory temperature (RT; 25 °C), the optical density at 695 nm was quantified using a UV–VIS spectrophotometer, and an analytical reference blank was also employed. As a standard, ascorbic acid was employed.

#### 2.4.2 DPPH assay for antioxidant potential

LRLME's efficacy in quenching DPPH radicals was analyzed following an accepted procedure from earlier studies using 2,2'-diphenyl-1-picrylhydrazyl (DPPH; Sigma-Aldrich) as the target radical [39], [40]. 1 mL of the LRLME extract or ascorbic acid was homogenized with 3 mL of methanolic DPPH solution (0.1 mM), allowing for assessing radical suppression potency. Thereafter, the reaction medium was stabilized at RT for 30 min under light-protected settings. Subsequently, the extract sample was spectrophotometrically analyzed at 517 nm, and the percentage inhibition of free radicals was inferred in line with the following equation:

Scavenging (%) = (A_c_ – A_s_) / A_c_ × 100

In this case, "A_c_" represents the control's absorbance (DPPH blank), and "A_s_" represents the tested sample's ultimate absorbance following a 30-min incubation period.

#### 2.4.3 ABTS test for antioxidant potential

The antioxidant potential of LRLME against the ABTS radical was evaluated in alignment with the earlier experiments reported by Re et al. (1999) [41]. To produce the ABTS radical cation, ABTS stock solution (7 mM) was infused with 2.45 mM potassium persulfate in equal volumes (1:1, v/v). To complete the reaction, the prepared mixture was retained at room temperature in the dark for 10–16 h. The optical absorbance of the diluted ABTS•+ solution was read at 734 nm and recorded as 0.70 ± 0.02. Next, 3.0 mL of the ABTS•+ solution was placed into 1 mL of the sample solutions prepared at varying concentrations, and the combined mixture was subjected to vortex mixing for homogenization. Following a 6-min retention, 734 nm readings were recorded. The positive control in this experiment was ascorbic acid. The formula, as follows, was used to determine the scavenging rate:

Scavenging (%) = (A_c_ – A_s_) / A_c_ × 100

In this case, "A_c_" = control absorbance (ABTS blank), and "A_s_" = sample's ultimate absorbance following 6 -min of incubation.

### 2.5 Brine shrimp lethality bioassay

The cytotoxic potential of LRLME was evaluated against *Artemia salina* using a one-day *in vivo* bioassay, implemented with a prior protocol with minor adjustments [42]. Using a serial dilution technique, the extract was assessed at doses of 5–800 μg/mL. The stock solution was formulated by solubilizing 5 mg of extract in 1 mL of distilled water, yielding a concentration of 5 μg/μL. Aliquots were delivered to test tubes holding 5 mL of simulated seawater, each hosting ten live nauplii and a parallel control group. Following incubation for 24 h, the vials were examined under a magnifier, and the viable nauplii per test tube were enumerated and noted. The mortality percentage for each concentration was subsequently computed, and the LC_50_ value was computed by using Probit analysis according to an established protocol [43].

### 2.6 Assessment of antiproliferative efficacy in an animal model

#### 2.6.1 Ethical clearance and animal use

Four groups of adult male Swiss albino mice (22–27 g) were established, with six animals included in each group. The mice were housed in the animal center of Rajshahi University under the Biochemistry & Molecular Biology Department. Animals were maintained within well-aerated polypropylene cages, subjected to a 12-h light/dark photoperiod, and placed under controlled laboratory conditions (temperature: 23‒27 °C and humidity: 50‒60%). Before the commencement of the experiment, the mice were supplied with freely accessible standard diet and drinking water, and were permitted seven days to adapt to the laboratory conditions. Animal experimentation was performed in line with global ethical standards and institutional regulations, following the approval and guidance of the Institutional Animal, Medical Ethics, Bio-Safety, and Bio-Security Committee (IAMEBBC), Institute of Biological Sciences, University of Rajshahi, Bangladesh (License No: 208/320/(69)IAMEBBC/IBSc).

#### 2.6.2 Harvesting of EAC cells

The first batch of EAC cells used in this study was acquired from the Indian Institute for Chemical Biology (IICB), Kolkata, India. They were then kept alive in the lab using the technique outlined by Habib et al. [44]. Ehrlich Ascites Carcinoma (EAC) in its ascitic form was generated through intraperitoneal administration of a tumor cell suspension into mice. The progressive growth of tumor cells within the peritoneal cavity led to the formation of a characteristic milky-white ascitic fluid containing abundant large, rounded tumor cells. For experimental purposes, ascitic fluid was harvested from EAC-bearing Swiss albino mice during the respective logarithmic phases of tumor cell growth. Ascitic tumor cells were harvested using a sterile 3 mL syringe fitted with a 20-gauge needle. The freshly collected fluid was diluted in normal saline (0.98% NaCl), and the tumor cell concentration was adjusted to approximately 1 × 10^6^ cells/mL through haemocytometric counting. Viability of the harvested cells was determined using a 0.4% trypan blue exclusion assay, and only preparations with more than 90% viable cells were considered suitable for transplantation. Subsequently, 0.1 mL of the standardized suspension, containing 1 × 10^5^ viable tumor cells, was administered intraperitoneally to each Swiss albino mouse. Throughout the procedure, rigorous aseptic techniques were employed to ensure sterility and experimental reliability. Thus, they were kept alive in the lab via every two weeks intraperitoneal inoculation of 10^5^ cells for every mouse.

#### 2.6.3 Evaluation of anticancer potentiality

The anticancer potential of LRLME was assessed by tracking EAC cell proliferation, tumor burden, and lifespan of tumor-bearing mice, including hematological profiles.

#### 2.6.4 Investigation of cell growth inhibitory effects

To evaluate the inhibitory effect of LRLME on tumor cell proliferation, four groups of Swiss albino mice (22–27 g, n = 6 per group) were utilized. On the initial day (Day 0), Swiss albino mice were inoculated intraperitoneally with 1.36 × 10^6^ EAC cells to establish tumors for therapeutic testing. Treatment regimens were initiated 24 h thereafter and maintained for a five-day course. Group I represented the untreated EAC-bearing control, Groups II and III were injected with LRLME at intraperitoneal doses of 20 and 40 mg/kg, respectively, and Group IV comprised normal healthy animals without tumor induction or intervention (the negative group/normal). The selected doses were chosen based on preliminary toxicity assessment and published literature. LRLME exhibited relatively low toxicity in the brine shrimp lethality assay (LC_50_ = 661.278 μg/mL). In addition, previous studies on *L*. *rubiginosa* demonstrated significant biological activity at doses of 20 mg/kg and 100 mg/kg in experimental mice (p < 0.05), including an *in vivo* study employing 40 mg/kg [30], [45]. In addition, a published EAC study (Same tumor-model precedent) reported significant tumor growth inhibition at doses of 25 and 50 mg/kg in mice [46]. Therefore, 40 mg/kg was selected as the higher dose, and 20 mg/kg as the lower dose to evaluate dose-dependent antineoplastic activity while minimizing the risk of treatment-related toxicity.

On day 6, three animals each from Groups I–III were sacrificed, and intraperitoneal tumor cells were aspirated using 0.98% isotonic saline and mixed with 0.4% trypan blue dye. Cell viability (Viable and non-viable cells) was determined using the trypan blue exclusion method in a haemocytometer by using a microscope, where viable cells excluded the dye while non-viable cells appeared blue. The total number of viable EAC cells was calculated and used as an indicator of tumor cell proliferation. The number of viable cells in the treatment group was compared with the number of viable cells counted in the EAC control. The positive effect of the LRLME against EAC cell-bearing mice was further verified by monitoring the change in hematological parameters. The remainder of the animals were monitored for hematological investigations, with treatment extended for 10 days in the intervention groups [47]. Alterations in haematological parameters with the growth of the tumor in the control & treatment groups were monitored by comparing with the haematological parameters of the negative control group/normal mice. The tumor cell growth inhibition (%) was determined by employing the formulae:

% Cell Growth Inhibition = (1 – MVT_t_/MVT_c_) × 10

NB: MVT_t_ represents the treated group's mean viable cell counts, and MVT_c_ represents the control group's mean viable cell counts.

#### 2.6.5 Monitoring tumor weight & lifespan extension

Tumor regression and host survival were assessed in accordance with a modified [48] procedure. Swiss albino mice were stratified into four groups of six animals, each inoculated with EAC cells on day 0. Drug administration commenced 24 h post-cell inoculation and was maintained over a 10-day regimen. Tumor progression was indirectly evaluated from daily increments in body weight. Mortality was recorded for each group, and longevity was expressed as mean survival time (MST, days). Percentage increase in lifespan (ILS%) relative to untreated control animals was determined using the formulae:

MST = Σ survival time (days) of each mouse in a group/total number of mice

% ILS = (MST of treated group / MST of control group – 1) × 100

#### 2.6.6 Hematological studies

To determine the hematopoietic effects of LRLME in neoplastic conditions, 24 Swiss albino mice were stratified into four groups (n = 6). Each mouse was challenged intraperitoneally with EAC cells (1.36 × 10^6^/mouse). Twenty-four hours post-inoculation, intraperitoneal administration of LRLME was initiated and continued for 10 consecutive days. The treatment design was as follows:

**Group I (EAC Control):** Tumor-bearing mice, untreated.
**Group II (LRLME–Low):** Tumor-bearing mice, treated with LRLME at 20 mg/kg/day.
**Group III (LRLME–High):** Tumor-bearing mice, treated with LRLME at 40 mg/kg/day.
**Group IV (Normal):** Healthy control animals without tumor induction or drug exposure.

On day 12 following EAC cell inoculation, blood samples were withdrawn from the tails of three randomly selected mice from each experimental group. Hematological parameters, including WBC, RBC, and hemoglobin, were quantified using a hemocytometer with specific diluting fluids, in accordance with the established methodology of Shafiq et al. [48].

### 2.7 Qualitative phytochemical identification

#### 2.7.1 Sample preparation

After dissolving the plant extract in methanol, a vortex mixer was used to completely mix the mixture. The liquid sample that had dissolved was then passed through a syringe filter (0.22 μm). Ultimately, the extract's prepared sample was loaded into the GC-MS separation column.

#### 2.7.2 GS-MS-based phytochemicals identification

GC–MS profiling was conducted via splitless injection of 1 μL of sample into a Shimadzu QP2010 instrument equipped with a capillary column interfaced with a mass detector. The system was operated under the following optimized conditions: oven temperature of 100 °C, pressure of 89.7 kPa, total flow rate of 12.2 mL/min, column flow rate of 1.20 mL/min, linear velocity of 40.8 cm/s, and purge flow rate of 5.0 mL/min. Mass spectral acquisition was programmed from 5.00 to 50.00 min. The chromatographic peaks were verified by matching their spectral profiles with reference spectra available in the NIST library [49].

### 2.8 Virtual screening for anticancer drug candidates (*In silico* evaluation)

#### 2.8.1 Retrieval, cleaning, and optimization of protein and ligand structures

The 3D coordinates of EGFR (PDB ID: 1M17) were retrieved from the RCSB Protein Data Bank repository (https://www.rcsb.org/) [50], [51]. The X-ray diffraction data provided a high-resolution crystal structure (2.16 Å), defining the atomic and molecular geometry of the protein [50]. The protein preprocessing was accomplished using Maestro v11.4, which employed the built-in Protein Preparation Wizard at default settings [52]. During protein preprocessing, bond orders were corrected, hydrogens were added, missing side chains were reconstructed, and crystallographic water molecules were excluded. The 3D conformations of candidate ligands were obtained from PubChem (https://pubchem.ncbi.nlm.nih.gov/) and refined using the LigPrep module of Maestro v11.4 [52]. Ultimately, energy refinement of the prepared receptor and ligand models was achieved with the OPLS3e force field [53].

#### 2.8.2 Ligand-protein binding affinity through docking studies

Molecular docking was implemented through the Glide program of Schrödinger Suite, wherein bioactive GC–MS compounds were evaluated for their affinity and binding orientations toward the chosen protein target [54]. Docking experiments were executed in SP (standard precision) mode using the OPLS3e force field to ensure the accuracy of binding predictions [53], [55]. The co-crystallized native 4-anilinoquinazoline inhibitor erlotinib was used to define the active binding pocket of the target protein, and a receptor grid was generated accordingly. The docking grid was constructed around the binding site residues, with dimensions centered at X = 13.334, Y = 14.058, and Z = 69.482. Following docking, the interaction energies between the protein and ligands were estimated, and the specific binding residues, along with intermolecular bonds, were examined using Maestro visualization tools.

#### 2.8.3 Pharmacokinetic profiling of selected compounds

Pharmacokinetics (PK) investigates the fate of a drug within an organism, focusing on how it enters the body, how it is transported and transformed, and how it is removed, with respect to both the intensity and time course of action. Pharmacokinetic profiling is a core step in computational drug development, ensuring that potential compounds exhibit favorable ADME characteristics for clinical translation [56]. To investigate the ADME and drug-likeness profiles of the chosen molecules, SwissADME (http://www.swissadme.ch/index.php), a freely accessible web server, was employed [57]. The platform allows for systematic evaluation and prediction of multiple pharmacokinetic parameters of the selected ligands.

#### 2.8.4 *In silico* toxicity assessment

Early toxicity profiling is a critical component of rational drug discovery strategies [58]. Analysis of toxicological effects often targets mutagenic, carcinogenic, and other pertinent endpoints, which can be systematically quantified or described qualitatively [59]. In this study, the toxicity profiles of the selected phytocompounds, encompassing various toxicity endpoints, were deduced using the ProTox-3.0 server (https://tox.charite.de/protox3/) [60].

#### 2.8.5 Molecular dynamics simulation

The dynamic behavior and stability of the protein–ligand complex under physiological conditions were analyzed using a 100 ns MD simulation. The simulations were executed using the Desmond software suite, integrated within Schrödinger's computational platform [61]. Protein–ligand complex configurations derived from docking studies were prepared using the Protein Preparation Wizard, followed by solvation with the simple point-charge (SPC) water model in an orthorhombic periodic boundary box to accommodate the system volume [62]. For physiological relevance, the solvated complex was supplemented with Na^+^ and Cl^−^ ions to attain a 0.15 M salt concentration, followed by energy minimization and system equilibration using OPLS3e [63]. Temperature and pressure were controlled at 300 K and 1.01325 bar, respectively, using the constant pressure–constant temperature (NPT) ensemble [64]. After system equilibration, production trajectories were recorded every 100 ps, with an energy convergence criterion of 1.2 kcal/mol, and dynamic stability metrics such as RMSD, RMSF, rGyr, and SASA were computed to evaluate complex stability [32].

### 2.9 Statistical analysis

Experimental parameters, including tumor weight, lifespan, cell growth inhibition, and hematological measurements, were represented as mean ± SEM. One-way ANOVA followed by Duncan's multiple range test or an independent *t*-test was performed in GraphPad Prism 8.0.2, considering p < 0.05 as statistically significant.

## 3 Results

### 3.1 Polyphenol quantification of LRLME

#### 3.1.1 Estimation of bioactive polyphenolic compounds in LRLME

Quantification of phenolics, flavonoids, flavonols, and proanthocyanidins in LRLME was performed using standard calibration plots of gallic acid, catechin, and quercetin, with results summarized in Table 1. This evaluation demonstrated a notably high polyphenolic content in LRLME.

**Table 1:** Quantitative profiling of phytochemicals in LRLME.

Catalog of Polyphenol Constituents	Polyphenol Yield
Phenolics	61.292 ± 0.902 mg GAE/g of LRLME
Flavonoids	130.718 ± 1.862 mg CAE/g of LRLME
Flavonols	356.111 ± 1.156 mg QUE/g of LRLME
Proanthocyanidins	617.490 ± 0.707 mg CAE/g of LRLME

The reported values correspond to the mean ± SEM, with n = 3 experimental replicates (see Fig. 1).

### 3.2 LRLME's antioxidant potential

#### 3.2.1 Total antioxidant capacity (TAC)

In Fig. 2 (A), the graphical outcome of the TAC assay for LRLME is presented. Standard ascorbic acid showed higher antioxidant activity than LRLME at all tested concentrations. The spectrophotometric reading indicated 0.340 absorbance for LRLME versus 1.482 for ascorbic acid at 160 μg/mL. LRLME showed a progressive enhancement in antioxidant activity as the extract dose increased.

**Fig. 1:**
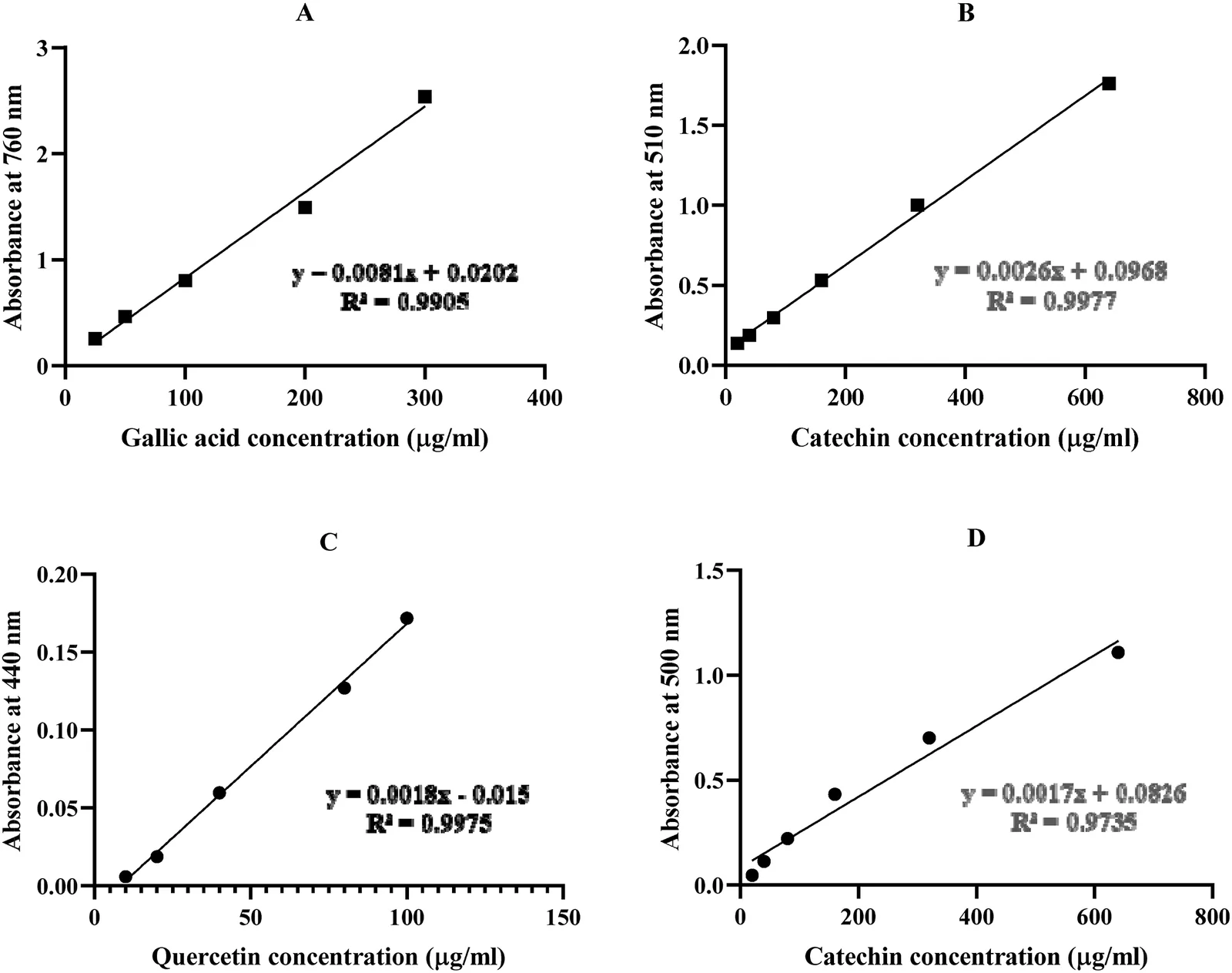
The analytical determination of phenolics, flavonoids, flavonols, and proanthocyanidins in LRLME was based on calibration standards: (A) gallic acid, (B) catechin, (C) quercetin, and (D) catechin. Quantitative data are given as mean ± SEM (n = 3). LRLME: *L. rubiginosa* leaves methanolic extract.

**Fig. 2:**
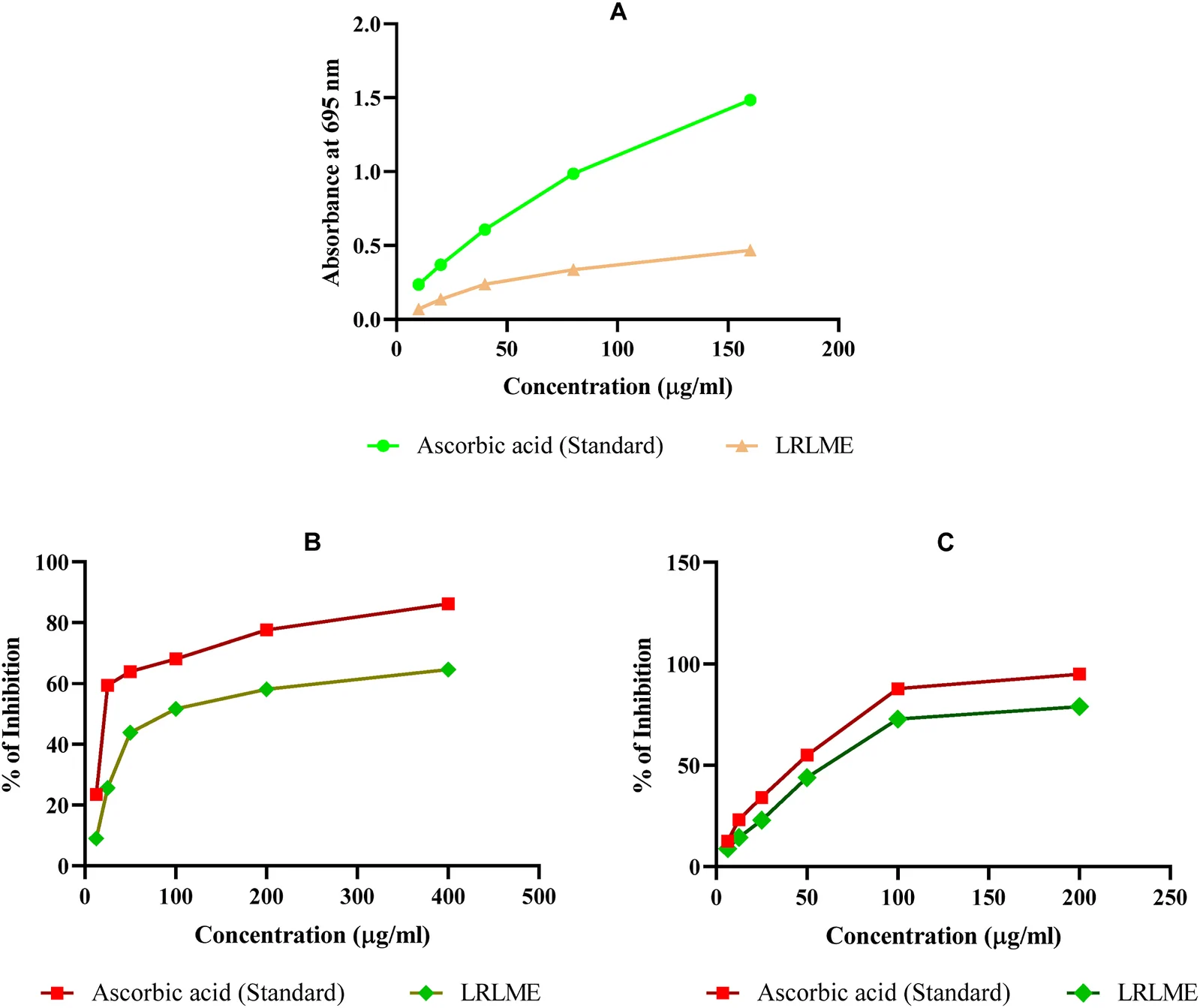
(A) Total antioxidant capacity measurement of LRLME and ascorbic acid. (B) DPPH radical scavenging performance. (C) ABTS radical scavenging performance. Percent inhibition of DPPH and ABTS radicals was used to express the findings. Observed values are shown as average values ± SEM for three experimental repeats. LRLME: *L. rubiginosa* leaves methanolic extract.

#### 3.2.2 Measurement of free radical neutralization through DPPH assay

Fig. 2 (B) illustrates the progressive increase in LRLME's free radical scavenging activity with concentration. At all evaluated doses, ascorbic acid maintained superior free radical-scavenging capacity compared to LRLME, with maximum inhibition percentages of 86.24% and 69.30%, respectively, at 400 μg/mL. Determined IC_50_ values for the DPPH assay were 11.647 μg/mL (ascorbic acid) and 168.718 μg/mL (LRLME); lower IC_50_ signifies more effective disruption of radical chain reactions.

#### 3.2.3 Measurement of free radical neutralization through ABTS assay

The enhanced chemical reactivity of ABTS•+ allows radical quenching via electron transfer, providing a mechanistic contrast to the hydrogen atom donation characteristic of DPPH. LRLME's capacity to neutralize ABTS radicals was further assessed. Results of ABTS•+ radical scavenging assay are illustrated in Fig. 2 (C). With 200 μg/mL, the highest concentration used in the assay, the inhibition percentages of LRLME and AA were 78.947 ± 0.570 and 94.978 ± 1.859, respectively. The radical quenching activity of AA and LRLME increased in accordance with concentration. The IC_50_ value of standard AA and LRLME was 62.574 and 91.702 μg/mL, respectively.

### 3.3 Assessment of LRLME-induced mortality in *Artemia salina*

Increasing the concentration of LRLME resulted in higher mortality of *Artemia salina* nauplii, with the peak lethality recorded at 800 μg/mL. Probit regression analysis yielded an LC_50_ of 661.278 μg/mL (Fig. 3), derived from the linear regression model Y = 0.0765x – 0.5679, indicating minimal cytotoxicity.

**Fig. 3:**
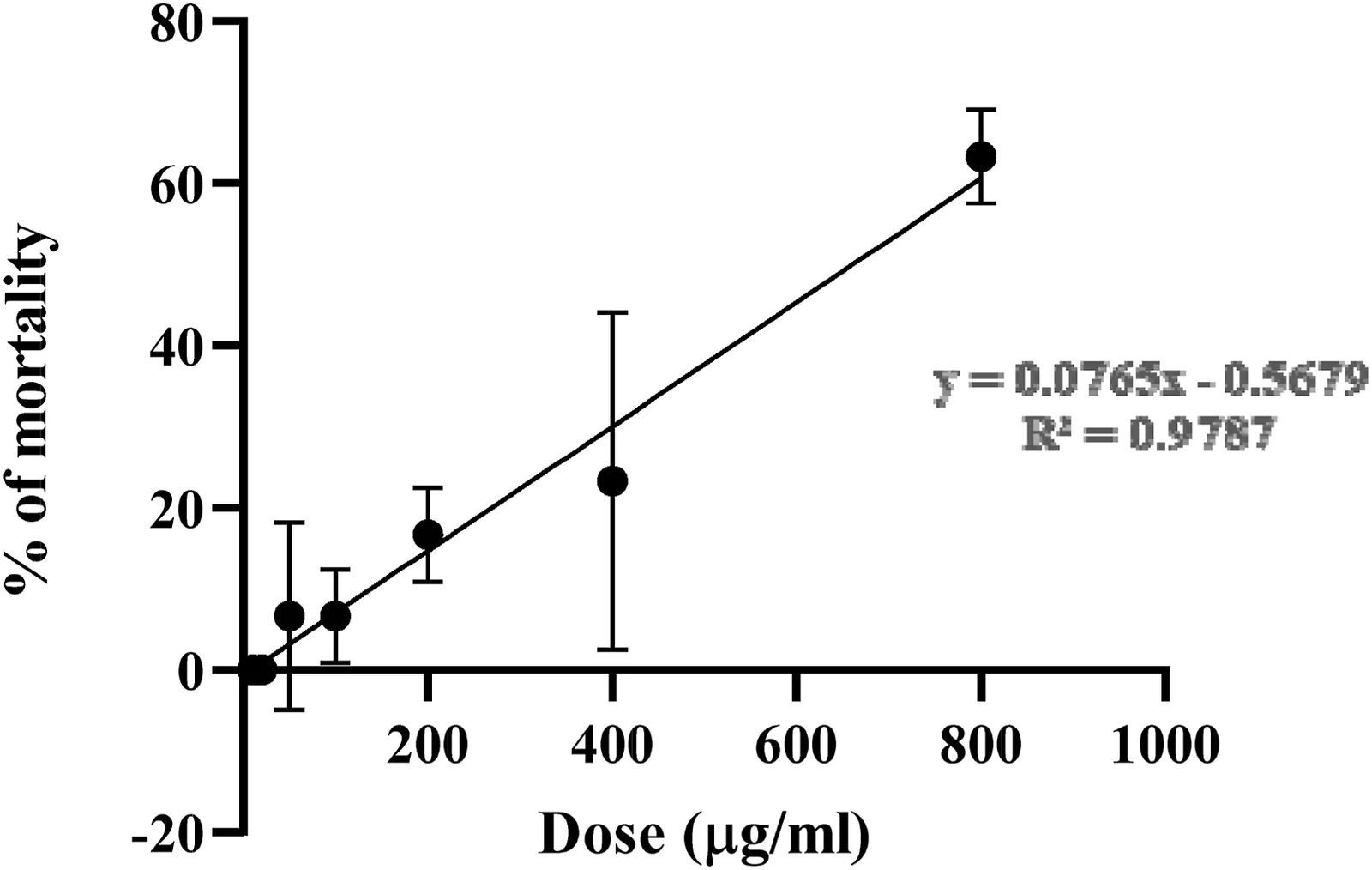
*Artemia salina* larvae mortality to multiple LRLME concentrations following 24-h exposure. Each value corresponds to mean ± SEM, calculated from ten independent observations. LRLME: *L. rubiginosa* leaves methanolic extract.

### 3.4 Assessment of LRLME on tumor cell proliferation (*In vivo*)

#### 3.4.1 Effects of LRLME on EAC cells growth inhibition

LRLME exhibits significant cell growth inhibition activity because we found that both doses of LRLME significantly decreased the count of viable cellular units (Fig. 4). Mean counts of surviving tumor cells in the untreated control group were (4.48 ± 0.196) × 10^7^ cells/mL, while administration of LRLME at 20 and 40 mg/kg (i.p.) significantly lowered these numbers to (3.013 ± 0.071) × 10^7^ and (2.047 ± 0.078) × 10^7^ cells/mL, respectively (Table 2). The antiproliferative effect of LRLME was most pronounced at 40 mg/kg (i.p.), achieving 54.31% inhibition, whereas 20 mg/kg induced 32.74% suppression (Table 2).

**Fig. 4:**
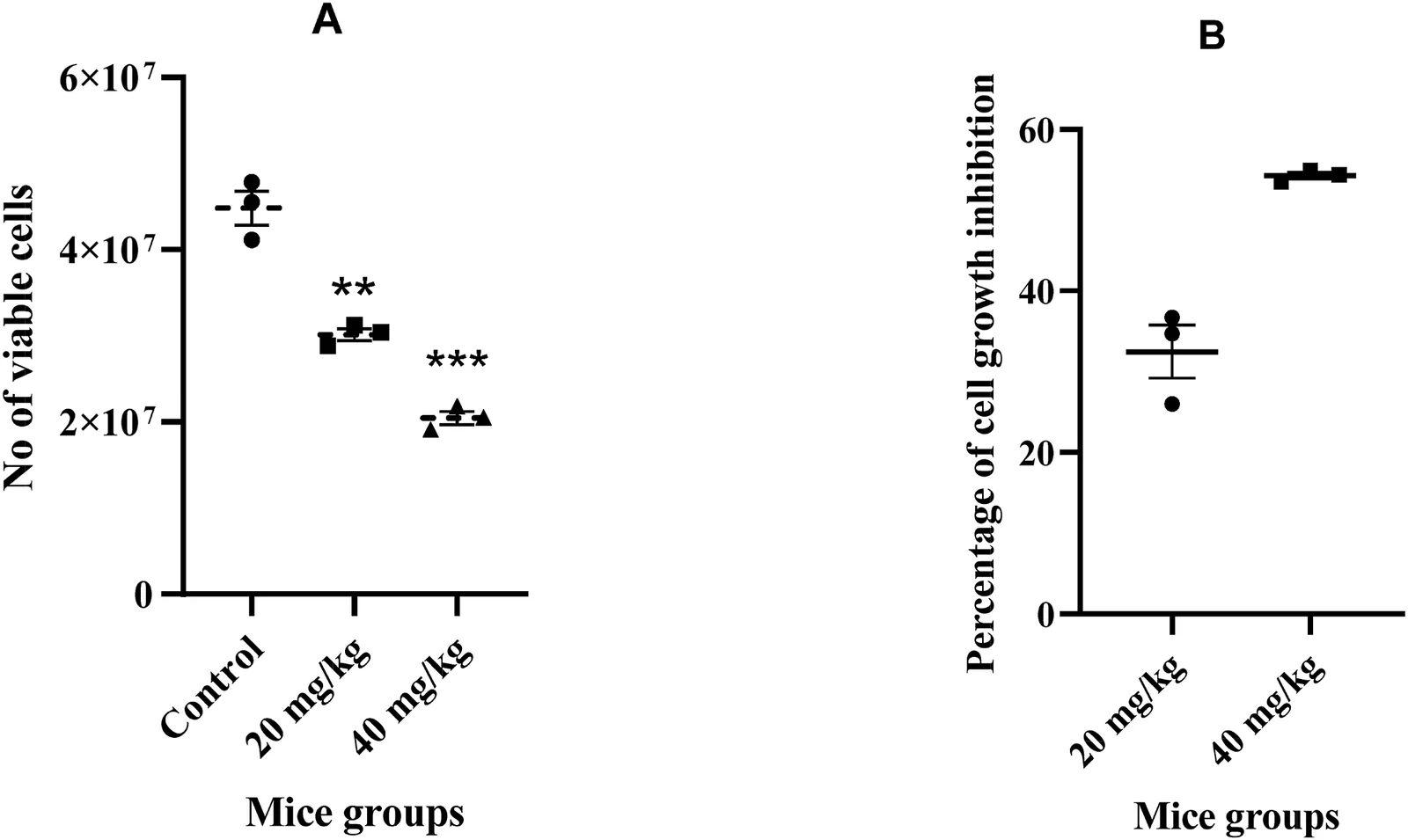
Effects of LRLME on EAC cell proliferation suppression. (A) LRLME-mediated suppression of viable EAC cells in tumor-bearing mice and (B) Relative percentage inhibition of EAC cell growth at 20 and 40 mg/kg in comparison to control mice. Individual data points represent values obtained from each mouse, and horizontal bars denote the mean ± SEM (n = 3). Results show that treatment with LRLME significantly affected EAC-bearing mice versus controls (**p < 0.01, ***p < 0.001). LRLME: *L. rubiginosa* leaves methanolic extract; EAC: Ehrlich Ascites Carcinoma; Low dose: 20 mg/kg/day of LRLME; High dose: 40 mg/kg/day of LRLME.

**Table 2:** Assessment of LRLME on survival time, percentage of life span, and tumor weight in the EAC-inoculated mice.

Categorization of experimental mice	Surviving EAC cells per mouse at day 6 post-tumor induction	Cell proliferation inhibitory percentage	Survival duration (MST, days)	Relative life span enhancement (% ILS)	Mean increase in tumor weight in EAC-inoculated mice at day 20.
Control (EAC cell-bearing mice)	(4.48 ± 0.196) × 10^7^	-----	24.667 ± 1.453	-----	32 ± 0.577
LRLME (20 mg/kg)	(3.013 ± 0.071) × 10^7^**	32.74	37.333 ± 2.186**	51.35	14 ± 1*
LRLME (40 mg/kg)	(2.047 ± 0.078) × 10^7^***	54.31	48.667 ± 1.202***	97.30	7 ± 0.577**

NB: Results are presented as mean ± SEM; *, **, *** mark significance at p < 0.05, p < 0.01, and p < 0.001 versus control. LRLME: *L. rubiginosa* leaves methanolic extract; EAC: Ehrlich Ascites Carcinoma; MST: Mean survival time; % ILS: Percentage increase in lifespan.

#### 3.4.2 Determination of LRLME impact on tumor growth, MST, and percentage life span enhancement in EAC tumor model

The effect of LRLME (injected i.p. at 20 and 40 mg/kg body weight) on tumor weight resulting from tumorigenesis has been summarized in Table 2 and Fig. 5. Significant tumor suppression was observed in mice with EAC-induced tumors following LRLME administration. With reference to the control cohort, which showed a 37 g increase in body mass by day 22, LRLME treatment effectively curtailed body weight gain (Table 2). By day 22, tumor weight in mice receiving LRLME at 20 and 40 mg/kg (i.p.) increased by 15 g and 8.33 g, respectively, reflecting the approximately twofold greater antitumor effect of the 40 mg/kg dose (Table 2). In control animals with EAC, the MST was limited to 24.667 ± 1.453 days, while LRLME treatment prolonged survival significantly to 37.333 ± 2.186 days at 20 mg/kg and 48.667 ± 1.202 days at 40 mg/kg (see Fig. 6), reflecting 51.35% and 97.30% enhancements in lifespan. Mice administered 40 mg/kg LRLME showed a 97.30% increase in survival, significantly greater than the 51.35% lifespan extension observed with 20 mg/kg treatment.

**Fig. 5:**
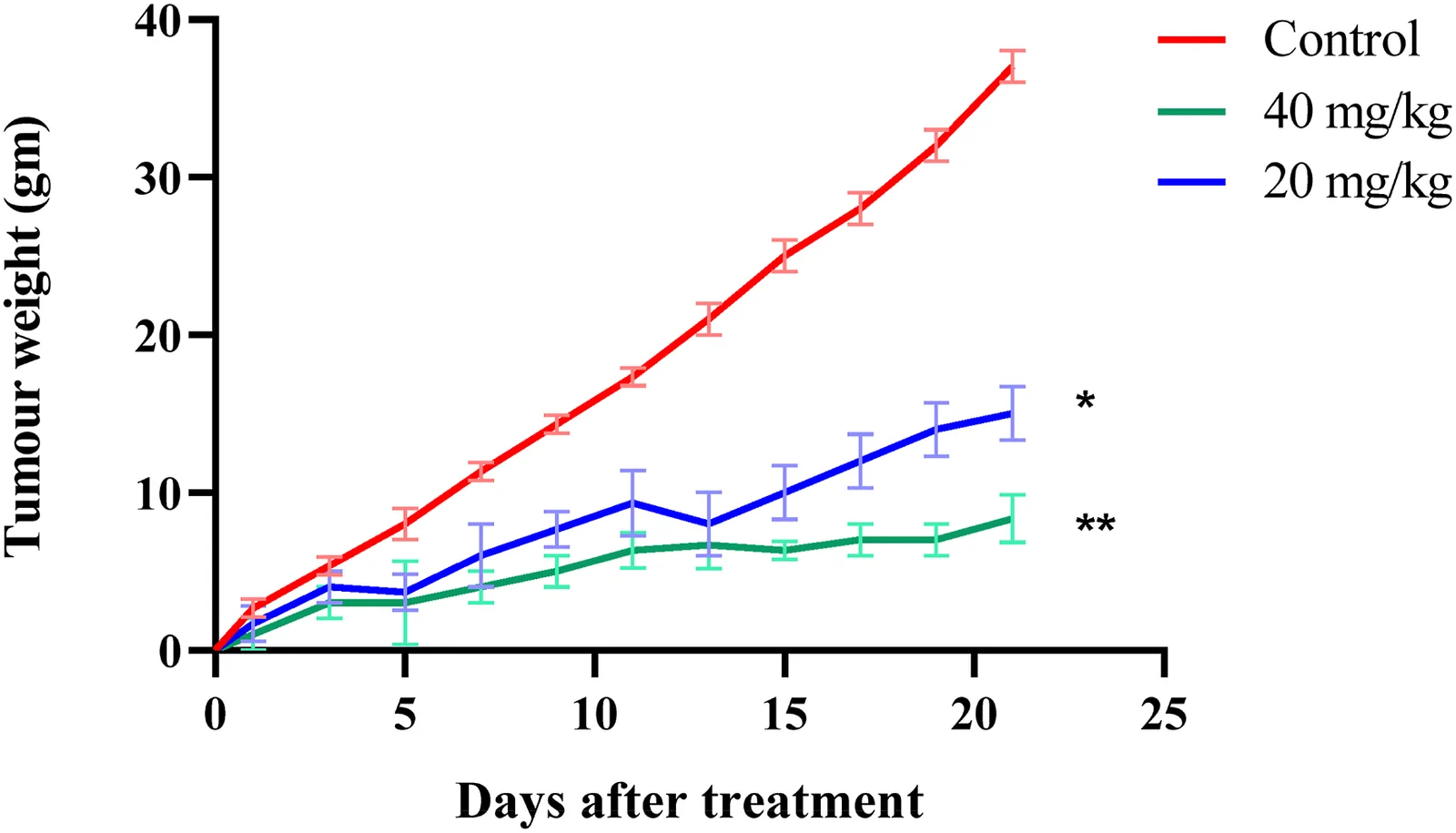
LRLME-induced tumor weight suppression in EAC-challenged mice. Mean ± SEM values are displayed, with differences considered statistically relevant at *p < 0.05 and **p < 0.01 against EAC-bearing controls. LRLME: *L. rubiginosa* leaves methanolic extract; Low dose: 20 mg/kg/day of LRLME; High dose: 40 mg/kg/day of LRLME.

**Fig. 6:**
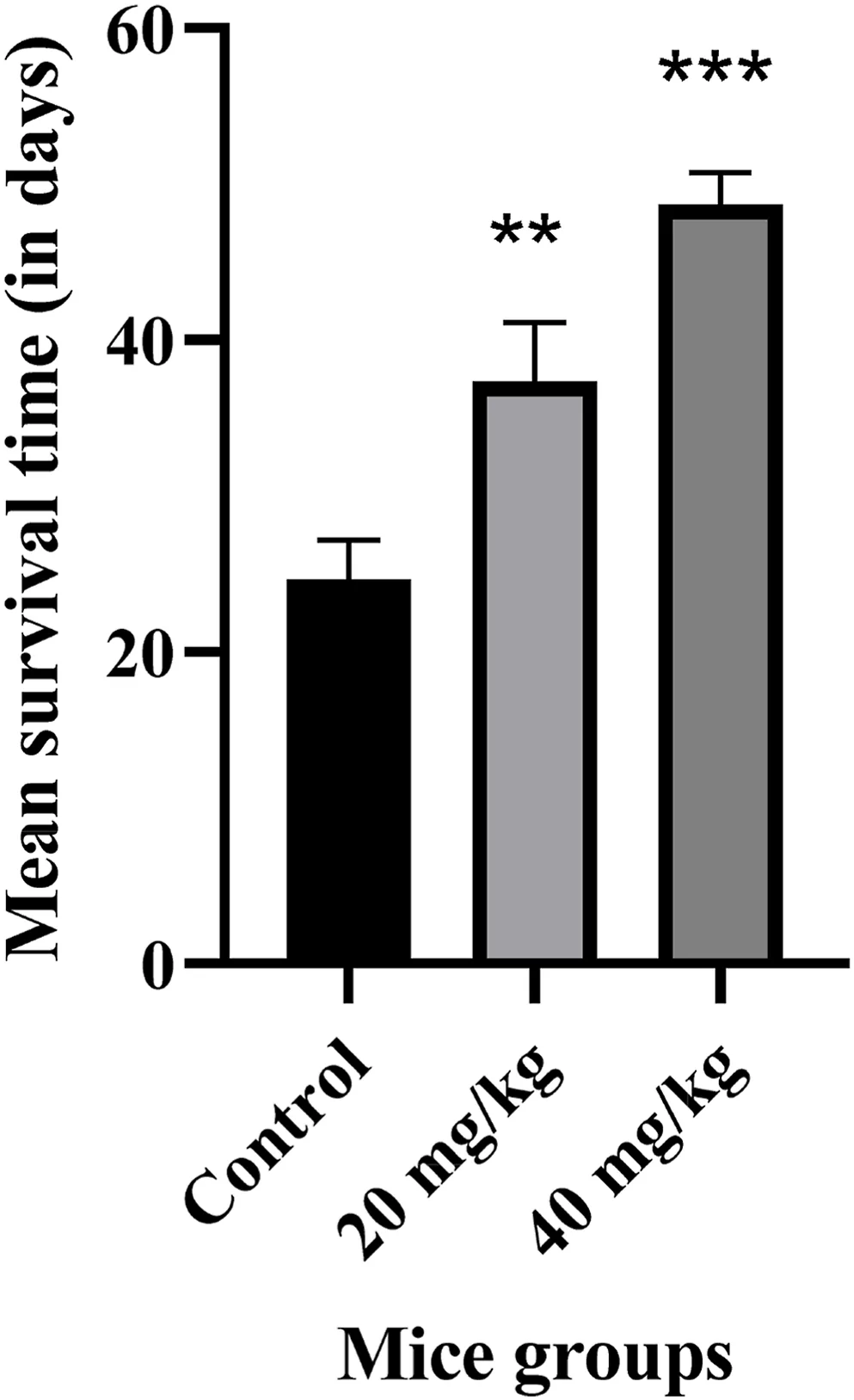
Effect of LRLME treatment in extending the survival of tumor-bearing mice. Mean ± SEM is shown for all data, with **p < 0.01 and ***p < 0.001 reflecting differences from control animals. LRLME: *L. rubiginosa* leaves methanolic extract; Low dose: 20 mg/kg/day of LRLME; High dose: 40 mg/kg/day of LRLME.

#### 3.4.3 Determination of LRLME-induced modulation of blood parameters in EAC tumor model

Tumor development in EAC-bearing mice led to notable changes in hematological indices compared to healthy controls. EAC tumor development in mice resulted in marked fluctuations in RBC, WBC, and Hb percentages (Table 3). Nevertheless, treatment involving LRLME at 40 mg/kg (i.p.) restored these deteriorated hematological parameters (e.g.*,* RBC, WBC, Hb) significantly toward normal values in relation to the control EAC tumor-bearing cohort (Table 3).

**Table 3:** Influence of LRLME on erythrocyte, leukocyte, and hemoglobin levels in tumor-bearing mice at day 12 post-inoculation.

Mice groups	Blood profile indicators		
	RBC cells/ml	WBC cells/ml	% of Hb (gm/dl)
Normal Mice	(7.173 ± 0.098) × 10^9^	(0.967 ± 0.218) × 10^7^	9.033 ± 0.088
Control Mice (EAC bearing)	(4.887 ± 0.032) × 10^9^	(8.467 ± 1.049) × 10^7^	4.1 ± 0.115
LRLME (20 mg/kg)	(5.497 ± 0.078) × 10^9^**	(2.333 ± 0.437) × 10^7^**	5.767 ± 0.145***
LRLME (40 mg/kg)	(6.090 ± 0.115) × 10^9^***	(1.533 ± 0.067) × 10^7^**	7.433 ± 0.067****

NB: Findings are summarized as mean ± SEM of three observations (n = 3); with significance versus control indicated by Significance is marked by *, **, ***, and **** corresponding to p < 0.01, p < 0.001, and p < 0.0001 versus control. LRLME: *L. rubiginosa* leaves methanolic extract; EAC: Ehrlich Ascites Carcinoma; RBC: Red blood cell; WBC: White blood cell; Hb: Hemoglobin.

### 3.5 GS-MS-based phytochemicals identification

Gas chromatography–mass spectrometry (GC-MS) examination of *L. rubiginosa* revealed a total of 99 distinct peaks. Based on retention time (RT), peak area %, and an empirical formula, each peak corresponded to a unique compound. Over the 40-min analysis period, the methanolic extract of *L. rubiginosa* leaves yielded 54 non-redundant putatively identified compounds, as seen in Fig. 7 and outlined in Table 4. The majority of these compounds were eluted within the retention time occurring within 4.0–39.0 min.

**Fig. 7:**
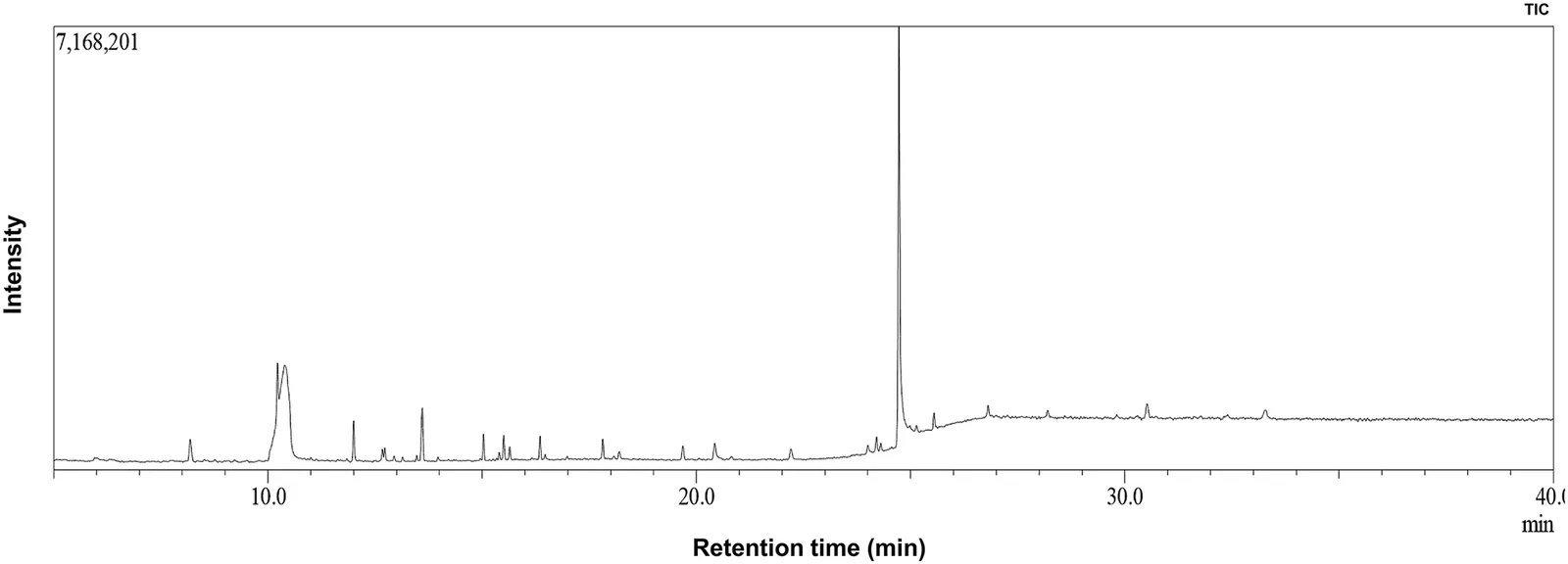
GC-MS chromatogram of metabolite annotation of *L. rubiginosa* leaves methanolic extract.

**Table 4:** Catalog of GC–MS–identified bioactive constituents from *L. rubiginosa* leaves methanolic extract.

Peak	R. Time (min)	Compound Name	CID
1.	5.272	3-Azabicyclo[3.2.2]nonane	9240
2.	5.272	2-Heptanamine, 5-methyl-	541442
3.	6.179	Urea, butyl-	11595
4.	6.546	Paradrine	3651
5.	6.546	Norpseudoephedrine	441457
6.	6.546	Octodrine	10982
7.	7.346	Benzeneethanamine, 2,5-dimethoxy-.alpha.-methyl-	62787
8.	8.240	Propanenitrile, 3-amino-2,3-di(hydroxymino)-	136259682
9.	9.845	Benzeneethanamine, 4-methoxy-α.-methyl	3016422
10.	10.225	.alpha.-Methyl mannofuranoside	143402
11.	12.335	Phytol	5280435
12.	12.455	2-Octynoic acid	21872
13.	12.724	2-Pentadecanone, 6,10,14-trimethyl-	10408
14.	12.936	Dibutyl phthalate	3026
15.	13.586	Hexadecanoic acid, methyl ester	8181
16.	13.812	7,9-Di-tert-butyl-1-oxaspiro(4,5)deca-6,9-diene	545303
17.	13.812	Butylated Hydroxytoluene	31404
18.	13.812	Cystine	67678
19	13.812	(−)-Norephedrine	10297
20.	13.966	1,2-Benzenedicarboxylic acid, butyl octyl ester	66540
21.	13.994	Propanamide	6578
22.	13.994	Phenylephrine	6041
23.	14.408	Cyclopropyl carbinol	75644
24.	14.408	Cathinone	62258
25.	15.066	Glutaraldehyde	3485
26.	15.103	Amphetamine	3007
27.	15.103	1,2-Ethanediamine, N-(2-aminoethyl)-	8111
28.	15.103	10-Undecenal	8187
29.	15.103	Benzenemethanol, .alpha.-[(methylamino)methyl]-	913
30.	15.103	3-Decyne	75425
31.	15.644	Methyl stearate	8201
32.	16.550	Arginine	6322
33.	16.550	Folic Acid	135398658
34.	16.550	Carbromal	6488
35.	16.550	Butanamide	10927
36.	16.939	Heptanal	8130
37.	16.939	2H-Azepin-2-one, hexahydro-1-methyl-	17369
38.	17.660	3,6-Dimethylpiperazine-2,5-dione	97704
39.	17.660	2-Methylaminomethyl-1,3-dioxolane	541754
40.	17.930	9-Octadecenamide, (Z)-	5283387
41.	20.433	1-Octadecanamine, N-methyl-	75539
42.	20.433	Glycerol 1-palmitate	14900
43.	20.433	2-Piperidinone, 1-methyl-	13603
44.	21.607	d-Alanine	71080
45.	23.800	Decanoic acid, 2,3-dihydroxypropyl ester	92926
46.	23.800	cis-1,2-Cyclohexanediol	92903
47.	23.800	Octadecanoic acid, 2,3-dihydroxypropyl ester	24699
48.	24.727	1,4-Benzenedicarboxylic acid, bis(2-ethylhexyl) ester	22932
49.	24.727	13-Docosenamide, (Z)-	5365371
50.	24.727	Hexadecenoic acid, Z-11-	5312414
51.	24.727	1-Eicosanol	12404
52.	24.727	Erucic acid	5281116
53.	24.727	Oleic Acid	445639
54.	25.055	4-Tetradecanol	33740

NB: CID: Compound Identifier; R. Time: Retention Time.

### 3.6 *In silico* analysis

#### 3.6.1 Protein ligand binding affinity through molecular docking

Docking-based analysis was undertaken to predict the strength of protein–ligand binding interactions, serving as a preliminary step in drug design and validation. The epidermal growth factor receptor (PDB ID 1M17) and the obtained phytocompounds were subjected to docking analysis to interpret the interaction potential between the modeled protein structure and photochemical ligands, and the docking score with their GI absorption, BBB permeability, and Lipinski violations are listed in the Supplementary Table 1. The compounds from LRLME, namely phenylephrine (CID 6041), cathine (CID 441457), and (−)-Norephedrine (CID 10297), showed the highest energy scores of −6.249 kcal/mol, −6.474 kcal/mol, and −6.644 kcal/mol, respectively (Table 5). Based on the docking score and pharmacokinetics properties like GI absorption, BBB permeability, and Lipinski violations, three compounds (CID 6041, CID 10297, and CID 441457) were picked, using −6.00 kcal molmol^−1^ as the cut-off binding energy value. The docking score of the standard drug bleomycin (CID 5360373) was −5.671 kcal molmol^−1^ (Table 5), which is less stable than our selected compounds. The final three compounds were prioritized for subsequent analyses.

**Table 5:** Molecular docking scores of the EGFR protein (PDB; 1M17) and selected three compounds with the control drug are presented.

Compound Name	Compound CID	Docking Score	GI Absorption	BBB Permeability	Lipinski Violation
(−)-Norephedrine	10297	−6.644	High	Yes	Yes, 0 Violations
Cathine	441457	−6.474	High	Yes	Yes, 0 Violations
Phenylephrine	6041	−6.249	High	Yes	Yes, 0 Violations
Bleomycin (control)	5360373	−5.671	Low	No	No, 3 violations

NB: EGFR: Epidermal Growth Factor Receptor; CID: Compound Identifier; GI: Gastrointestinal; BBB: Blood- Brain Barrier.

#### 3.6.2 Protein–ligand interaction analysis

The three compounds with the strongest protein–ligand interactions were taken forward for additional evaluation. The Maestro module of the Schrödinger suite was used to depict the molecular interactions of the top-scoring compounds with the target protein, as depicted in Fig. 8. Various non-covalent interactions, including hydrogen bonds, electrostatic contacts, polar interactions, and hydrophobic contacts, were identified between the ligands and the receptor. The compound CID 6041 interacted through a hydrogen bond with ASP831, polar bonds (THR766, GLN767, ASN818, and THR830), and hydrophobic interactions (LEU694, PHE699, VAL702, TYR703, ALA718, ILE720, ALA739, MET742, CYS751, LEU753, LEU764, ILE765, LEU768, MET769, CYS773, VAL819, LEU820, PHE832) with distinct amino acid residues of the targeted protein. The compound CID 10297 also interacted through a hydrogen bond ASP831, polar bonds (ASN818 and THR830), and hydrophobic interactions (LEU694, PHE699, VAL702, TYR703, ALA718, ILE720, ALA739, MET742, CYS751, LEU753, LEU764, ILE765, LEU768, MET769, CYS773, VAL819, LEU820, PHE832) with the active pocket amino acid residues. Whereas the compound CID 441467 showed stronger interactions than the other compounds, which included are hydrogen bonds (THR830 and ASP831), polar bonds (THR701, THR766, GLN767, ASN818), and hydrophobic interactions (LEU694, PHE699, VAL702, TYR703, ILE720, ALA719, MET742, CYS751, LEU753, LEU764, ILE765, LEU768, CYS773, LEU820, PHE832) within the target protein's active site. In comparison to the lead candidates, the standard drug bleomycin (CID 5360373) showed fewer interactions, which are polar bonds (GLN107, HIS136, THR142), and hydrophobic interactions (PRO103, LEU106, ALA110, LEU130, ALA133, VAL134, LEU135, LEU138), but no hydrogen bonds with the amino acid residues. All three ligands engaged with multiple residues of the target protein, which are illustrated in Fig. 8 and listed in Table 6.

**Fig. 8:**
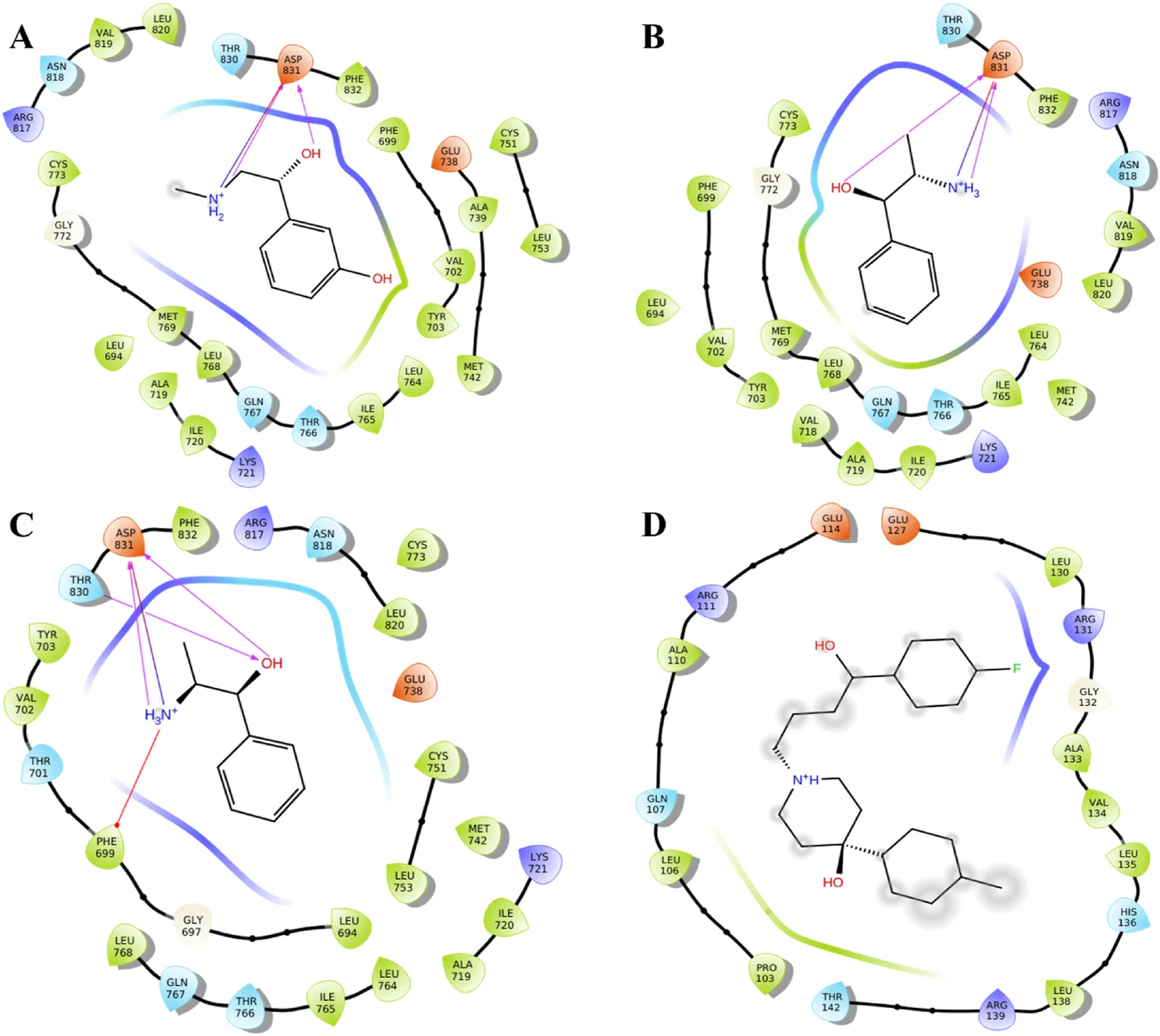
Interaction between the EGFR (PDB; 1M17) and the selected three compounds with the control is represented in 2D format. Representing phenylephrine (A), (−)-norephedrine (B), cathine (C), and bleomycin (control) in the active pocket of the protein. EGFR: Epidermal Growth Factor Receptor.

**Table 6:** Molecular docking score and amino acid-binding residues between EGFR and the selected three compounds, along with the control drug.

CID	Docking score (kcal/mol)	H-bond	Polar bond	Hydrophobic bond
6041	−6.249	ASP831	THR766, GLN767, ASN818, THR830	LEU694, PHE699, VAL702, TYR703, ALA718, ILE720, ALA739, MET742, CYS751, LEU753, LEU764, ILE765, LEU768, MET769, CYS773, VAL819, LEU820, PHE832
10297	−6.644	ASP831	ASN818, THR830	LEU694, PHE699, VAL702, TYR703, ALA718, ILE720, ALA739, MET742, CYS751, LEU753, LEU764, ILE765, LEU768, MET769, CYS773, VAL819, LEU820, PHE832
441457	−6.474	THR830, ASP831	THR701, THR766, GLN767, ASN818	LEU694, PHE699, VAL702, TYR703, ALA719, ILE720, MET742, CYS751, LEU753, LEU764, ILE765, LEU768, CYS773, LEU820, PHE832
5360373 (control)	−5.574	-	GLN107, HIS136, THR142	PRO103, LEU106, ALA110, LEU130, ALA133, VAL134, LEU135, LEU138

NB: EGFR: Epidermal Growth Factor Receptor; CID: Compound Identifier; CID 10297: (−)-Norephedrine; CID 441457: Cathine; CID 6041: Phenylephrine; CID 5360373: Bleomycin (control).

#### 3.6.3 Assessment of pharmacokinetic properties

The pharmacokinetic properties, including ADME (absorption, distribution, metabolism, and excretion), are affected by the route of drug administration and the physiological status of the organism, as well as by the chemical properties of the active substance. These factors play a key role in anticipating and preventing clinical trial failures that might otherwise have been unforeseen. The selected compounds were therefore assessed for their pharmacokinetic profiles. In particular, compounds with PubChem CID: 6041, 10297, 441457, and 5360373 were assessed for physicochemical properties, lipophilicity, solubility, GI absorption, drug-likeness (Lipinski's Rule of Five), and synthetic feasibility (Table 7). All ligands showed desirable pharmacokinetic features, pointing to a low failure risk in the later stages of clinical development.

**Table 7:** Pharmacokinetic properties, including physicochemical properties, lipophilicity, water solubility, GI absorption, drug-likeness, and synthetic accessibility of the three chosen compounds.

	CID Number	CID 6041	CID 10297	CID 441457	CID 5360373
	Compound Name	Phenylephrine	(−)-Norephedrine	Cathine	Bleomycin
Physicochemical Properties	MW (g/mol)	167.21 (g/mol)	151.21 (g/mol)	151.21 (g/mol)	1415.6 (g/mol)
	Heavy atoms	12	11	11	57
	Aromatic heavy atoms	6	6	6	11
	Rotatable bonds	3	2	2	20
	H-bond acceptors	3	2	2	20
	H bond donors	3	2	2	14
Lipophilicity	Log Po/w(MLOGP)	0.65	1.26	1.26	−7.5
Water Solubility	Log S (ESOL)	−0.85	−1.57	−1.57	1.05
Pharmacokinetics	GI absorption	High	High	High	Low
Drug likeness	Lipinski; Violation	Yes, 0 violations	Yes, 0 violations	Yes, 0 violations	No, 3 violations
Medicinal Chemistry	Synthetic accessibility	1.62	1.62	1.62	7.70

NB: EGFR: Epidermal Growth Factor Receptor; CID: Compound Identifier; GI: Gastrointestinal; MW: Molecular Weight; CID 10297: (−)-Norephedrine; CID 441457: Cathine; CID 6041: Phenylephrine; CID 5360373: Bleomycin (control).

#### 3.6.4 Assessment of toxicity

The toxicity of a chemical compound is the extent to which it can be harmful to a living organism. It can interfere with the release of essential enzymes and can significantly alter the function of a specific organ after single or prolonged exposure. Sometimes substances that are intended to be drug candidates can have harmful effects on both human and animal bodies, including cytotoxicity, immunotoxicity, mutagenicity, or organ toxicity. The toxicity of the three compounds chosen for this study, phenylephrine (CID: 6041), (−)-norephedrine (CID: 10297), cathine (CID: 441457), and the standard drug bleomycin (CID 5360373) was assessed. The LD_50_ value represents the minimum dose at which substances can be considered as lethal to 50% of animals tested. For instance, a substance with a low LD_50_ value is regarded as highly toxic. A material with a high LD_50_ value, on the other hand, is thought to be comparatively less toxic. Additionally, the toxicity class indicates the probability that a substance will cause harm to the body based on its toxicity profile. The toxicity profiles of all three compounds were excellent, and they are all classified as slightly toxic by toxicity class 3. The standard drug bleomycin (CID 5360373) exhibited a favorable toxicity profile compared to lead compounds, and it is classified as non-toxic by toxicity class 4. The toxicity profiles of lead compounds and the standard drug are listed in Table 8.

**Table 8:** Assessment of organ toxicity and toxicity endpoints of the three chosen compounds.

Endpoint	Target	CID; 6041	CID; 10297	CID; 441457	CID 5360373
Organ toxicity	Hepatotoxicity	Inactive; 0.96	Inactive; 0.93	Inactive; 0.93	Inactive; 0.59
	Neurotoxicity	Inactive; 0.53	Inactive; 0.50	Inactive; 0.50	Active; 0.66
	Respiratory toxicity	Active; 0.93	Active; 0.59	Active; 0.59	Active; 0.84
	Cardiotoxicity	Inactive; 0.69	Active; 0.58	Active; 0.58	Inactive; 0.59
Toxicity endpoints	Carcinogenicity	Inactive; 0.83	Inactive; 0.59	Inactive; 0.59	Inactive; 0.53
	Immunogenicity	Inactive; 0.79	Inactive; 0.99	Inactive; 0.99	Active; 0.99
	Mutagenicity	Inactive; 0.96	Inactive; 0.91	Inactive; 0.91	Active; 0.87
	Cytotoxicity	Inactive; 0.74	Inactive; 0.77	Inactive; 0.77	Inactive; 0.60
	LD_50_ (mg/kg)	120 (mg/kg)	150 (mg/kg)	150 (mg/kg)	800 (mg/kg)
	Toxicity class	3	3	3	4

NB: LD_50_: Lethal Dose, 50%; CID: Compound Identifier; CID 10297: (−)-Norephedrine; CID 441457: Cathine; CID 6041: Phenylephrine; CID 5360373: Bleomycin (control).

#### 3.6.5 Molecular dynamics simulation

MD simulation offers a computational setting and provides an artificial environment that could be implemented in drug design studies to evaluate the stability and receptor-ligand interactions at the molecular level based on a reference time. It creates a mimicked physiological condition that determines the structural transitions of the complex. Therefore, an MD simulation of 100 ns duration was executed in this study. The results of the MD simulation were inspected on the basis of RMSD, RMSF, rGyr, and SASA analysis.

##### 3.6.5.1 RMSD evaluation

RMSD is exploited to assess the average positional deviation of selected atoms over a defined time interval. RMSD shows how compactly the ligand's atoms bind to the protein's amino acid residues, and it helps to distinguish protein stability based on its average atomic fluctuations. To explore the conformational dynamics of the protein–ligand complexes involving CID: 441457, CID: 10297, CID: 6041, and the control compound CID: 5360373, MD simulations were run for 100 ns. The corresponding RMSD values are presented in Fig. 9 (A) and Supplementary Table 2. Regarding the ligand CID: 441457, the mean RMSD metric was around 8 Å, and the fluctuation was a little bit higher, but the stability was consistently maintained. For the compounds CID: 6041 and CID: 10297, the average fluctuation was around 7 Å and 12 Å, respectively. The fluctuations were observed to be higher between 40 and 70 ns, while the complex exhibited good stability during the remaining simulation period. Interestingly, the control drug CID: 5360373 showed an average RMSD around 2.5 Å, which was better than the apo protein and lead compounds, which means it was compactly bound throughout the simulation time. The overall results underpin that the control stability is better than that of druggable compounds, but the apo protein, with a native ligand, had a fluctuation range around 6 Å, similar to the lead compounds, which means no issue about the stability of such compounds. Therefore, the compound CID: 441457 showed moderate stability among other compounds, so it can be a potential druggable compound.

**Fig. 9:**
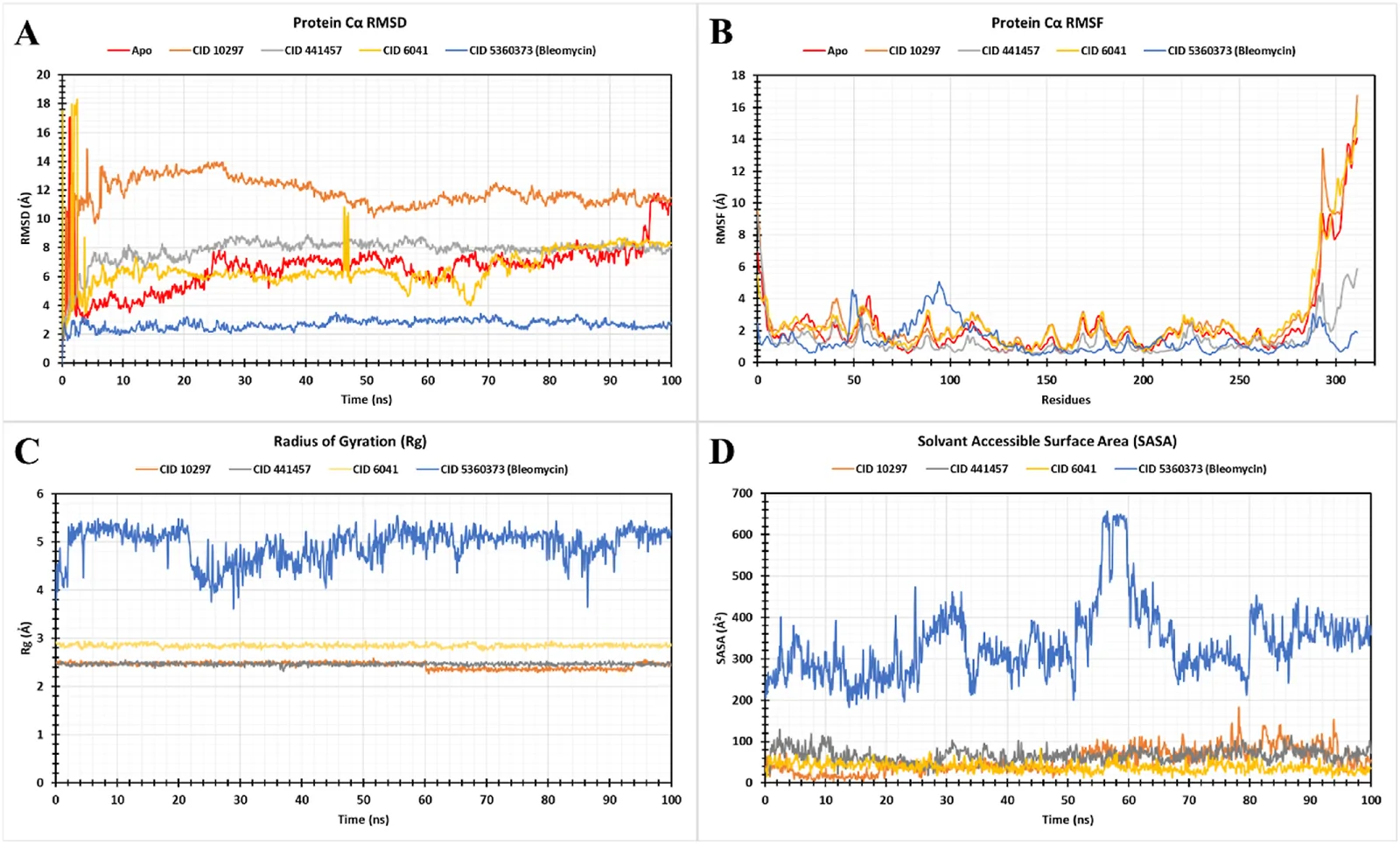
Molecular dynamics simulation outcomes of EGFR in complex with three chosen phytocompounds. (A) Representing the RMSD numerical findings of the EGFR in complex with the three lead phytocompounds. The druggable phytocompounds CID; 10297, CID; 441457, CID; 6041, and CID; 5360373 (control), forming a complex with the target protein, were portrayed by orange, gray, yellow, and blue, respectively, while the RMSD of native EGFR (PDB ID; 1M17) is displayed in red. (B) The RMSF values of EGFR in complex with the three selected compounds are presented. The RMSF profile of native EGFR (PDB ID: 1M17) is depicted in red, while the complexes with compounds CID: 10297, CID: 441457, CID: 6041, and the control CID: 5360373 are represented in orange, gray, yellow, and blue, respectively. (C) The radius of gyration (Rg) of the EGFR–ligand complexes (PDB ID: 1M17) was calculated over a 100 ns simulation. Rg values for compounds CID: 10297, CID: 441457, CID: 6041, and the control CID: 5360373 are represented in orange, gray, yellow, and blue, respectively. (D) Solvent-accessible surface area (SASA) was analyzed over a 100 ns simulation for the protein–ligand complexes. SASA values for compounds CID: 10297, CID: 441457, CID: 6041, and the control CID: 5360373 are visualized in orange, gray, yellow, and blue, respectively. RMSD: Root Mean Square Deviation; SASA: Solvent‒Accessible Surface Area; RMSF: Root Mean Square Fluctuation; Rg: Radius of Gyration; EGFR: Epidermal Growth Factor Receptor; CID: Compound Identifier; CID 10297: (−)-Norephedrine; CID 441457: Cathine; CID 6041: Phenylephrine; CID 5360373: Bleomycin (control).

##### 3.6.5.2 RMSF analysis

The Root Mean Square fluctuation is used to measure and characterize the shifts recorded in amino acid residues during protein and ligand interaction over a simulation timeframe. The compounds CID: 441457, CID: 6041, CID: 10297, and the control CID: 5360373 in complex with AKT (protein kinase B) were analyzed to assess changes in the protein's structural flexibility at specific residue positions during ligand binding, as illustrated in Fig. 9 (B). In this case, the compound CID: 441457 showed the average fluctuation range between 1.5 and 2.5 Å, which was similar to the control drug CID: 5360373. For instance, CID: 6041 and CID: 10297 showed moderate to low structural pliability, with measurements spanning between 2 and 4 Å and 3‒7 Å, respectively. Whereas the apo protein flexibility was measured at around 2‒5 Å, indicating the similarity of binding affinity between the native ligand and lead compounds (Supplementary Table 2). Overall fluctuations were observed primarily within residues 1–20 and 290–300, corresponding to the N/C termini. Contrastingly, the core regions of the protein exhibited stable interactions with the ligands. Based on the assessment, CID: 441457 is a promising lead compound due to its similar stabilizing effect to that of the control drug and consistent flexibility throughout the simulation time.

##### 3.6.5.3 RG analysis

The radius of gyration (Rg) is one of the important structural parameters that describes the spatial distribution of atoms around the mass center of the protein–ligand complex and is used to determine structural compactness and the fit of drugs with target receptors. Consequently, the Rg was also calculated for the stability of CID: 441457, CID: 10297, CID: 6041, and CID: 5360373 (control) during a 100 ns simulation time (Fig. 9 (C) and Supplementary Table 2), which is found to lie in the range of 2.31-2.55, 2.26-2.58, 2.74-2.95, and 3.60-5.54, respectively. The results imply that ligand binding does not induce substantial conformational alterations in the protein's active site, and the lower Rg value suggests that the ligands are compactly bound with their respective receptors. In comparison to the control drug, the three lead compounds showed consistent stability during the simulation timeframe, and the fluctuation rate was much lower than that of the standard drug.

##### 3.6.5.4 SASA analysis

The SASA is a computational method developed to assess the structure and function of biological macromolecules. The protein surface comprises amino acid residues that serve as active sites for ligand attachment and play a key role in determining whether a molecule is polar or non-polar. Thus, the solvent-like behavior of a molecule can be identified by SASA analysis, and the values for the docked protein (EGFR)–lead compound (CID: 441457, CID: 10297, and CID: 6041) complex were 17.76-129.73 Å^2^, 4.44-182.56 Å^2^, and 12.43-83.11 Å^2^, respectively, Fig. 9 (D) and Supplementary Table 2. The protein with higher SASA values has more surface area exposed to solvent, which may be more resistant to binding small molecules of drug, whereas a lesser SASA value implies a more compact protein configuration, which is amenable to drug binding. The three lead compounds' average SASA value fluctuations below 100 Å^2^ mean they are relatively stable over the 100 ns timeframe. However, in the case of the control drug, we found higher SASA value fluctuations, ranging from 182.64 to 657.29 Å^2^, which denotes lower stability than the lead compounds, Fig. 9 (D). The result underpins that our lead compounds have a compact structure with minimal exposure to the surrounding solvent.

## 4 Discussion

Cancer is a major global concern because of its rising incidence, high death rates, multifactorial nature, tumor heterogeneity, resistance to conventional therapies, complex treatment requirements, economic and psychological burden, and disparities in healthcare access. Moreover, during cancer control and treatment, severe toxicity and undesirable side effects associated with numerous modern synthetic medications result in compromised quality of life, reduced treatment adherence, and the development of secondary complications. Addressing the limitations of modern synthetic drugs necessitates the identification of novel, safer, less-toxic, and higher efficacy anticancer agents, ideally derived from natural, biocompatible sources like medicinal plants that are rich in diverse types of phytochemicals, like as polyphenols with multiple biological properties. Within this framework, this study effort is motivated to examine the antineoplastic potential of *L. rubiginosa*, a medicinal plant known for its ethnopharmacological uses.

Cancer development and progression are strongly influenced by oxidative stress, a condition characterized by excessive ROS accumulation that overwhelms the body's antioxidant defense systems. Oxidative stress frequently damages cellular DNA, leading to mutations and altered cellular signaling, which usually results in inconsistent cell proliferation as well as tumor formation [65]. Plant-derived natural compounds have multifaceted binding capabilities, which makes them potential drivers against multifactorial diseases like cancer, and over 60% of anti-cancerous drugs are derived from plants [66]. These secondary plant metabolites exert a significant and effective contribution to fighting cancer by exerting antioxidant activities for eliminating free radicals, promoting apoptosis, arresting the cell cycle, and inhibiting angiogenesis [67]. Empirical findings reveal that plant-derived bioactive constituents can mediate antineoplastic activity in mice challenged with EAC tumors, contributing to higher survival rates and extended lifespans [68], [69].

Amongst others, potent phytoconstituents like alkaloids and terpenoids exist, but the medicinal properties usually depend on their phenolic and flavonoid concentrations. Compared with many alternative solvents, methanol was selected as the extraction solvent because of its high polarity and well-documented effectiveness in extracting a broad spectrum of polar and moderately polar phytochemicals, including phenolics, flavonoids, alkaloids, terpenoids, and other bioactive secondary metabolites. In addition, methanol is compatible with subsequent GC–MS analysis and has been successfully employed in previous studies for extraction on *L. rubiginosa* [23], [35], [39], [70]. Our results demonstrated that the LRLME contains higher amounts of phenolics and flavonoid compounds (reported in GAE and CAE per gram of dry LRLME extract). However, these results varied from the prior findings [26], [71]. The reasons behind this variation in phenolic and flavonoid content of LRLME are assumed to stem from the variations in the agroclimatic environments of miscellaneous geographical positions, solvent polarity, and plant organ types. The higher number of polyphenols, including flavonoids, in LRLME may be responsible for exerting its antioxidant activity. It is thought that this activity is mainly caused by their redox properties; the redox capabilities of these molecules facilitate the capture and stabilization of reactive species [72]. To confirm its antioxidant potential, the *L. rubiginosa* leaves methanolic extract (LRLME) was analyzed using TAC, DPPH, and ABTS free radical scavenging assays. The TAC results revealed a pronounced concentration-dependent antioxidant effect. The phosphomolybdenum test is founded on molybdenum reduction by bioactive compounds, yielding a green molybdenum(V) complex with light absorption at 695 nm, in agreement with observations by Ref. [73]. As evidenced by this study, variations in the chemical composition of extracts may account for variations in their antioxidant ability. Phenolics and flavonoids were identified as the key chemical constituents of citrus antioxidant effects [73]. Therefore, the chemical composition, as well as phenolics and flavonoids, may underlie the total antioxidant activity of LRLME. DPPH and ABTS methods are frequently employed to investigate the radical scavenging properties of plant-derived extracts or synthetics that usually reflect the hydrogen-donating abilities of antioxidants. The DPPH and ABTS tests are based on reducing DPPH• and ABTS•+ in the presence of antioxidants, indicated through the color shift in DPPH (purple → yellow) and from (blue → white) in ABTS, which can be recorded spectrophotometrically [74]. The DPPH analytical procedure was employed to assess the antioxidant efficacy of LRLME in comparison to ascorbic acid. LRLME demonstrated strong free radical intercepting capability, yielding an IC_50_ of 168.718 μg/mL, while ascorbic acid displayed an IC_50_ of 11.647 μg/mL. These outcomes were consistent with the investigation (147.39 and 15.62 μg/mL) of *Oroxylum indicum* bark sample and ascorbic acid [75] and with the findings (30‒155 μg/mL for whole methanolic extracts and their respective solvent-partitioned fractions and 5.87 ± 0.21 to 27.50 ± 0.64 μg/mL for ascorbic acid) of [71]. ABTS analysis revealed IC_50_ values of 91.702 μg/mL for LRLME and 62.574 μg/mL for ascorbic acid, which were slightly comparable to the IC_50_ values of 98.07 and 23.20 μg/mL for the methanolic extract of *Oldenlandia corymbosa* Linn. and for ascorbic acid, respectively [26]. Differences in antioxidant activity among the extracts may arise from the qualitative and quantitative variations of the constituent antioxidant molecules. Moreover, IC_50_ values may differ from test to test and region to region. Consequently, the substantial presence of flavonoid and phenolic constituents in LRLME may underlie both its effective DPPH and ABTS radical neutralization and its antitumor potential.

Pharmacological and toxicological studies commonly use the Brine Shrimp Lethality Assay (BSLA) to track the cytotoxic, antimicrobial, or pesticidal potential of compounds prior to animal experimentation. This bioassay offers an effective, efficient, quick, and economically advantageous screening tool for determining the possible cytotoxic effects and lethality of test items, especially natural products, plant extracts, plus chemically synthesized molecules [76], [77], [78]. Based on toxicity assessments utilizing the BSLA, LRLME demonstrated a satisfactory safety profile with a higher LC_50_ value. Following this assay, LC_50_ values of 10 different medicinal plants were determined, 16.6–729.9 μg/ml, of which most, i.e., 9 medicinal plants, showed pronounced cytotoxic activity, as indicated by LC_50_ values in the range between 16.6 and 567.7 μg/ml, except the *Zingiber officinale,* with 729.9 μg/ml. Accordingly, from a toxicity perspective, the extract (LRLME) demonstrated minimal cytotoxicity toward Artemia, presenting an LC_50_ of 661.278 μg/mL, which indicates LRLME can be a safe and promising remedy for therapeutic use in traditional medicine.

Reliable criteria for assessing an anticancer medication include suppression of neoplastic cell proliferation, a decline in mean tumor volume, prolongation of host survival, and normalization of blood parameters like RBC, WBC, and Hb towards normal values [79]. Bone marrow suppression and anemia are primary complications associated with anticancer chemotherapy. The primary cause of anemia in tumor-induced mice is a diminishment in red blood cell count/hemoglobin levels. This arises from iron deficiency linked to hemolytic or bone marrow pathology [44]. Herein, we observed that LRLME treatment at 40 mg/kg (i.p.) markedly inhibited tumor growth and decreased tumor mass in EAC-bearing mice. This dosage also led to a significant extension of lifespan compared to untreated controls. Likely, administration of increased doses would further enhance survival duration. Lifespan extension is a vital measure in determining a plant's potential as an antiproliferative drug. Moreover, LRLME (40 mg/kg; i.p.) also restored the altered haematological parameters, i.e., RBC, WBC, and Hb, significantly towards near-normal levels, which signifies LRLME-mediated protection of the haemopoietic function in comparison to EAC-bearing animals. So, our findings support the compatibility of *L. rubiginosa* leaves as a natural anticancer agent.

In comparison to traditional methods, *in silico* analysis significantly improves the speed and cost-efficiency of using GC−MS-detected phytocompounds from the leaves of *L. rubiginosa* in drug discovery [80]. Our findings indicate that natural substances with dual- or multitarget inhibitory effects against EGFR and VEGFR-2, including hesperetin, gossypetin, quercetin, gallocatechin, and epigallocatechin, may be able to influence both of these signaling pathways [81]. According to this study, a multi-targeted molecule may offer as a potential therapy for cancer treatment. Each of the three compounds in the next stage of our *in silico* investigation showed molecular weights of less than 500 g/mol, efficient gastrointestinal absorption, aligning with Lipinski's guidelines, and were judged safe with respect to blood–brain barrier penetration. The three compounds also possessed practical physicochemical characteristics like water permeability, synthetic accessibility, and lipophilicity. These three hit phytoconstituents were opted for additional molecular dynamics simulation research due to their potential pharmacokinetic properties as prospective anticancer agents. MDS assessment confirmed that cathine, phenylpropanolamine, and cathinone exhibited robust and stable interactions with EGFR. Stability was evidenced by RMSD, RMSF, rGyr, SASA, protein–ligand contact analysis, and simulation snapshots, indicating their applicability as drug-like candidates for anticancer research. Root-mean-square deviation (RMSD) analysis was employed to monitor protein–ligand complex stability, whereas root-mean-square fluctuation (RMSF) was used to assess residue-specific structural flexibility within the selected macromolecules. The α-carbon (Cα) atoms served as key structural markers in the molecular dynamics simulations, with lower RMSD and RMSF values indicative of enhanced complex stability and reduced residue mobility. In this study, all complexes exhibited consistent and acceptable RMSD and RMSF profiles, confirming that the three lead phytocompounds maintained stable interactions within the EGFR active site. Radius of gyration (rGyr) analysis demonstrated that lower rGyr values corresponded to more compact protein–ligand assemblies, while higher rGyr values suggested partial ligand dissociation. The three selected ligands displayed lower rGyr peaks compared to the reference drug bleomycin, reflecting more tightly packed complexes. Similarly, solvent-accessible surface area (SASA) analysis documented that lower SASA values corresponded to ligands being closely engaged with surrounding amino acid residues, indicative of higher complex stability. Collectively, these molecular dynamics (MD) simulation parameters highlight the structural robustness and potential efficacy of the selected phytocompounds as promising EGFR-targeting anticancer agents, underscoring their relevance in rational drug design.

The compound cathine is primarily found as an alkaloid in the khat plant (*Catha edulis*), a shrub native to East Africa and the Arabian region [82]. In agreement with our results, literature indicates that the main psychoactive chemical entities of khat leaves are (−)-Cathinone [(S)-α-aminopropiophenone], (+)-Cathine [(1S,2S)-norpseudoephedrine], and (−)-Norephedrine [(1R,2S)-norephedrine], which exert sympathomimetic effects and contribute to its addictive potential [83]. Historically utilized as a psychoactive agent, *Catha edulis* has recently been identified to contain bioactive molecules capable of anticancer activity through mechanisms such as programmed cell death activation, tumor growth suppression effect, redox-balancing effect, immunomodulatory responses, and anti-inflammatory actions [84]. The previous study aligns with our findings as we identified similar phytocompounds from LRLME. Another promising lead phytochemical, (−)-norephedrine, is a sympathomimetic alkaloid that is found naturally in Ephedra species (e.g., *Ephedra sinica, Ephedra distachya*). (−)-Norepinephrine and cathine are similar compounds. They are both phenethylamines and amphetamine derivatives, and share the same core structure, differing only in the stereochemistry at the second carbon atom. Cathine is more commonly known as (+)-norpseudoephedrine, while (−)-norephedrine is a stereoisomer of cathine. Functioning chiefly as sympathomimetic agents, these compounds modulate sympathetic signaling pathways either through direct adrenergic receptor stimulation or by promoting endogenous catecholamine release [85]. Since cathine and (−)-norephedrine are both structurally similar compounds, their targets of action in the body are also similar. The third prioritized phytoconstituent, phenylephrine, represents an alkaloid-type metabolite, a class frequently associated with diverse therapeutic activities. Phenylephrine is used for its vasoconstrictive effects and its ability to decrease heart rate. It is also used as a nasal decongestant, as a previous study reported that liposomal formulations of phenylephrine nanoparticles demonstrated improved therapeutic outcomes by potentiating the antitumor activity of intratumoral chemotherapy against melanoma.

Computational evidence of the multi-target binding capacity of cathine, (−)-norephedrine, and phenylephrine supports our approach to experimentally evaluate the anticancer potential of *L. rubiginosa* leaf extract. EGFR is frequently mutated or overexpressed in several cancers, resulting in misdirected cellular signal relay networks, including PI3K/AKT, JAK/STAT, and MAPK/ERK, that promote cellular proliferation, survival, angiogenesis, and metastasis. Results from the growth inhibition assay confirmed a dose-dependent anticancer response, reinforcing that the phytochemical constituents of the extract, particularly cathine, (−)-norephedrine, and phenylephrine, are likely responsible for mediating the antitumor activity. Oxidative stress contributes significantly to oncogenesis by activating molecular pathways that facilitate the overproduction of ROS. Consequently, we assessed the antioxidant activity of the LRLME by evaluating their capacity to neutralize DPPH, ABTS, and TAC to demonstrate the impact of *L. ruiginosa* leaves on cancer. Dose-dependent antioxidant effects observed with the extract support the presence of potent bioactive molecules, thereby reinforcing our *in silico* findings and suggesting pharmacological relevance. As alkaloids, the three candidate phytochemicals align with earlier reports indicating that alkaloid-based compounds exhibit superior free radical scavenging and antioxidant properties, effectively neutralizing ROS and aiding in cancer treatment [86]. Integrated outcomes from both biological assays (*in vivo*) and computational analyses (*in silico*) reveal that *L. rubiginosa* leaves exert significant antineoplastic effects on EAC cells and may function as a prospective source of bioactive EGFR inhibitory molecules for future anticancer drug development applications.

*Limitations and Future Research Directions*: The extraction parameters, including maceration duration, were not systematically optimized. The extraction period was selected based on established laboratory protocols [39], [87]; therefore, the influence of different extraction time regimes on phytochemical yield and biological activity remains to be investigated. The evaluation of antitumor efficacy in the present study was primarily based on tumor cell count, survival analysis, and hematological parameters. However, gross photographic documentation of tumor progression, standardized tumor volume scaling, histopathological examination (e.g., H&E staining), and detailed morphological analyses of tumor tissues were not performed. The absence of these structural and cellular-level validations, together with the lack of mechanistic cell-death analyses such as Annexin V/PI staining and MTT assays, limits deeper interpretation regarding apoptosis induction, necrosis, angiogenesis suppression, restoration of normal tissue architecture, and the precise mode of tumor cell death following LRLME treatment. Although GC–MS profiling identified multiple phytoconstituents and *in silico* analyses (molecular docking, ADMET prediction, and molecular dynamics simulation) suggested strong binding affinities of selected lead compounds toward EGFR, these computational findings require experimental validation. Future studies should therefore incorporate Annexin V/PI staining, MTT assays, *in vitro* mechanistic investigations, gene and protein expression analyses of apoptotic and proliferative markers (e.g., caspases, Bax/Bcl-2 ratio, p53, Ki-67), and enzyme-based or receptor-specific inhibition assays to elucidate the mechanism of tumor cell death and confirm EGFR-targeted anticancer activity at the molecular level. Moreover, validation of the identified lead compounds through isolation, purification, and *in vivo* pharmacological testing is necessary to confirm their individual contributions to the observed antineoplastic effects. Comparative pharmacodynamic evaluation with standard chemotherapeutic agents and assessment in additional tumor models would further strengthen translational significance. Researchers have not yet published studies regarding the multi-targeted effect of these lead phytochemicals against cancer. Furthermore, these lead compounds possess diverse pharmacological applications, which may indicate potential for drug repurposing. Rigorous preclinical validation using advanced experimental model systems, followed by well-designed translational studies in cancer patients, is essential to verify their therapeutic effectiveness, safety, and clinical relevance. Collectively, addressing these limitations through integrated histopathological, molecular, toxicological, and translational investigations will provide a more comprehensive understanding of LRLME's anticancer potential and support its progression toward evidence-based drug development.

## 5 Conclusion

This study reveals that *Lepisanthes rubiginosa* leaves are natural sources of distinct phyto-bioactive compounds possessing strong antioxidant activity. Extract from these leaves has remarkable potential to inhibit Ehrlich Ascites Carcinoma (EAC) cell proliferation, reduce tumour weight, and extend the lifespan of mice inoculated with EAC cells. These findings reveal the correlation between antioxidant and antineoplastic attributes of *L. rubiginosa* leaves. Drug design, a rapidly evolving and effective approach, facilitates the identification of inhibitory compounds targeting specific proteins. We implemented a computer-assisted drug design (CADD) methodology to identify and characterize potential anticancer agents from *L. rubiginosa* leaves extract, aiming to identify novel and natural epidermal growth factor receptor (EGFR) inhibitors. The integrated CADD strategy, comprising docking studies, pharmacokinetic (ADMET) assessments, and molecular dynamics simulations, consistently pinpointed three candidate phytocompounds, CID: 10297 ((−)-norephedrine), CID: 441457 (cathine), and CID: 6041 (phenylephrine), which could significantly hinder the functional response of EGFR and consequently decrease cellular proliferation, survival, differentiation, and even reduce the risk of cancer formation. Based on simulation data, compound CID: 441457 (cathine) showed consistent binding stability in all assessed simulation parameters. We suggest these compounds for future anticancer drug design applications. Future studies should incorporate comprehensive histopathological and morphological validation, molecular and mechanistic confirmation of EGFR-targeted activity, isolation and structural characterization of the identified lead compounds, and comparative pharmacodynamic evaluation with standard chemotherapeutics to substantiate their translational and multi-targeted anticancer potential.

## CRediT authorship contribution statement

**Md. Taohid Hasan:** Writing – review & editing, Writing – original draft, Visualization, Methodology, Investigation, Formal analysis, Data curation, Conceptualization. **Md. Shakil Ahmed:** Writing – review & editing, Data curation. **Niloy Saha:** Writing – original draft, Methodology, Formal analysis. **Reaz Ahmmed:** Validation. **Md. Enamul Kabir Talukder:** Visualization, Investigation, Formal analysis. **Nafis Fuad Shahir:** Writing – review & editing. **Nazia Islam Rafi:** Validation. **Farzana Pervin:** Validation, Methodology. **Masudul Hasan Khan:** Resources. **Md. Rezaul Karim:** Resources. **Mohammad Amirul Islam:** Supervision, Project administration, Conceptualization.

## Ethics declaration

This study was conducted in accordance with the ARRIVE (Animal Research: Reporting of In Vivo Experiments) guidelines. This study was approved by the Institutional Animal, Medical Ethics, Bio-Safety, and Bio-Security Committee (IAMEBBC), Institute of Biological Sciences, University of Rajshahi, Bangladesh (Approval No. 208/320/(69)IAMEBBC/IBSc).

## Declaration of competing interest

The authors declare that they have no known competing financial interests or personal relationships that could have appeared to influence the work reported in this paper.

## Data Availability

Data will be made available on request.
