# Supplementary material for: Antineoplastic potential of Lepisanthes rubiginosa (Roxb.) Leenh. leaves extract against ehrlich ascites carcinoma: A multi‒approach study
Source: Biochem Biophys Rep. 2026 Oct 20;48:102835. doi: 10.1016/j.bbrep.2026.102835 (PMC13692136; doi:10.1016/j.bbrep.2026.102835)
Supplement: Supplementary file 1 [file mmc1.docx]

**Supplementary Table 1**: Molecular docking score of EGFR protein (PDB; 1M17) and phyto‒compounds of LRLME was identified through the GC-MS analysis.

| Compound Name | Compound CID | Docking Score | GI Absorption | BBB Permeability | Lipinski Violation |
| --- | --- | --- | --- | --- | --- |
|  |  |  |  |  |  |
| Folic Acid | 135398658 | -6.973 | Low | No | No; 2 Violations |
| (-)-Norephedrine | 10297 | -6.644 | High | Yes | Yes; 0 Violations |
| Cathine | 441457 | -6.474 | High | Yes | Yes; 0 Violations |
| Cathinone | 62258 | -6.289 | High | Yes | Yes; 0 Violations |
| N-Methyl-2-piperidone | 13603 | -6.257 | High | No | Yes; 0 Violations |
| Phenylephrine | 6041 | -6.249 | High | Yes | Yes; 0 Violations |
| N-Methylcaprolactam | 17369 | -6.201 | High | No | Yes; 0 violation |
| 7,9-Di-tert-butyl-1-oxaspiro[4.5]deca-6,9-diene-2,8-dione | 545303 | -5.725 | High | Yes | Yes; 0 violation |
| Bleomycin (control) | 5360373 | -5.671 | Low | No | No; 3 violations |
| Amphetamine | 3007 | -5.548 | High | Yes | Yes; 0 violation |
| Phenethylamine | 1001 | -5.417 | High | Yes | Yes; 0 violation |
| Carbromal | 6488 | -5.302 | High | No | Yes; 0 violation |
| alpha-D-Mannofuranoside, methyl | 143402 | -5.284 | Low | No | Yes; 0 violation |
| Hydroxyamfetamine | 3651 | -5.234 | High | Yes | Yes; 0 violation |
| Cathinone | 62258 | -5.007 | High | Yes | Yes; 0 violation |
| Cyclopropylmethanol | 75644 | -4.781 | High | No | Yes; 0 violation |
| 1,2-Cyclohexanediol, cis- | 92903 | -4.703 | High | No | Yes; 0 violation |
| Butyl octyl phthalate | 66540 | -4.689 | High | Yes | Yes; 1 violation |
| 1,3-Dioxolane-2-methanamine, N-methyl- | 541754 | -4.339 | High | No | Yes; 0 violation |
| 3-Azabicyclo[3.2.2]nonane | 9240 | -4.076 | High | No | Yes; 0 violation |
| Diethylenetriamine | 8111 | -3.989 | High | No | Yes; 0 violation |
| Glyceryl Monostearate | 24699 | -3.977 | High | No | Yes; 0 violation |
| Glyceryl Monostearate | 24699 | -3.977 | High | No | Yes; 0 violation |
| Erucamide | 5365371 | -3.629 | Low | No | Yes; 1 violation |
| Phenylpropanolamine | 10297 | -3.608 | High | Yes | Yes; 0 violation |
| 2,3-Dihydroxypropyl hexadecanoate | 14900 | -3.56 | High | Yes | Yes; 0 violation |
| Cystine | 67678 | -3.536 | Low | No | Yes; 0 violation |
| Octodrine | 10982 | -3.442 | High | Yes | Yes; 0 violation |
| 2,3-Dihydroxypropyl hexadecanoate | 14900 | -3.283 | High | Yes | Yes; 0 violation |
| Cathine | 441457 | -3.26 | High | Yes | Yes; 0 violation |
| Glutaral | 3485 | -3.249 | High | Yes | Yes; 0 violation |
| Propionamide | 6578 | -3.185 | High | No | Yes; 0 violation |
| 2-Cyano-N-hydroxy-2-hydroxyimino-acetamidine | 136259682 | -3.172 | Low | No | No; 2 violations |
| N-Butylurea | 11595 | -3.138 | High | No | Yes; 0 violation |
| L-arginine | 6322 | -3.121 | Low | No | Yes; 0 violation |
| 5-Methyl-2-heptanamine | 541442 | -3.121 | High | Yes | Yes; 0 violation |
| Heptanal | 8130 | -3.055 | High | Yes | Yes; 0 violation |
| Glyceryl Monostearate | 24699 | -2.969 | High | No | Yes; 0 violation |
| Phenylephrine | 6041 | -2.786 | High | No | Yes; 0 violation |
| Dibutyl Phthalate | 3026 | -2.694 | High | Yes | Yes; 0 violation |
| Cystine | 67678 | -2.455 | Low | No | Yes; 0 violation |
| D-Alanine | 71080 | -2.189 | High | No | Yes; 0 violation |
| 3-Decyne | 75425 | -1.975 | Low | Yes | Yes; 1 violation |
| Diethylenetriamine | 8111 | -1.954 | High | No | Yes; 0 violation |
| 2-Octynoic acid | 21872 | -1.909 | High | Yes | Yes; 0 violation |
| Hexahydrofarnesylacetone | 10408 | -1.418 | High | No | Yes; 1 violation |
| (Z)-11-hexadecenoic acid | 5312414 | -1.309 | High | Yes | Yes; 0 violation |
| Phytol | 5280435 | -0.779 | Low | No | Yes; 1 violation |
| Oleic Acid | 445639 | -0.698 | High | No | Yes; 1 violation |
| Oleamide | 5283387 | -0.522 | High | Yes | Yes; 1 violation |
| Glyceryl 1-caprate | 92926 | -0.281 | High | Yes | Yes; 0 violation |
| 4-Tetradecanol | 33740 | -0.216 | High | Yes | Yes; 0 violation |
| Methyl palmitate | 8181 | 0.005 | High | Yes | Yes; 1 violation |
| N-Methyloctadecylamine | 75539 | 0.206 | Low | No | Yes; 1 violation |
| Methyl Stearate | 8201 | 0.236 | High | No | Yes; 1 violation |
| 1-Eicosanol | 12404 | 0.569 | Low | No | Yes; 1 violation |
| 10-Undecenal | 8187 | 1.803 | High | Yes | Yes; 0 violation |

**Supplementary Table 2.** The three lead compounds, together with the control drug Bleomycin, produced various parameters, including the maximum, minimum, and average values, over the course of the 100 ns molecular dynamics simulation.

| **Parameter** | **Value** | **Apo** | **CID 10297** | **CID 441457** | **CID 6041** | **CID 5360373 (Bleomycin)** |
| --- | --- | --- | --- | --- | --- | --- |
| **Protein Cα RMSD** | H. RMSD (Å) | 17.044 | 14.865 | 8.903 | 18.286 | 3.459 |
|  | L. RMSD (Å) | 1.432 | 1.544 | 1.742 | 1.943 | 1.587 |
|  | A. RMSD (Å) | 6.608 | 11.781 | 7.807 | 6.591 | 2.685 |
| **Protein Cα RMSF** | H. RMSD (Å) | 14.078 | 16.763 | 9.224 | 15.651 | 5.053 |
|  | L. RMSD (Å) | 0.597 | 0.748 | 0.578 | 0.611 | 0.480 |
|  | A. RMSD (Å) | 2.298 | 2.636 | 1.455 | 2.604 | 1.361 |
| **Radius of gyration** | H. RMSD (Å) | N/A | 2.586 | 2.551 | 2.953 | 5.547 |
|  | L. RMSD (Å) | N/A | 2.267 | 2.314 | 2.742 | 3.608 |
|  | A. RMSD (Å) | N/A | 2.438 | 2.464 | 2.847 | 4.944 |
| **Solvent-accessible surface**  **area** | H. RMSD(Å^2^) | N/A | 182.567 | 129.738 | 83.116 | 657.299 |
|  | L. RMSD(Å^2^) | N/A | 4.448 | 17.760 | 12.538 | 182.642 |
|  | A. RMSD (Å^2^) | N/A | 53.286 | 66.619 | 38.498 | 336.896 |

H. - Highest, L.- Lowest, A. - Average
